# Mining belt foreign body detection method based on YOLOv4_GECA model

**DOI:** 10.1038/s41598-023-35962-3

**Published:** 2023-06-01

**Authors:** Dong Xiao, Panpan Liu, Jichun Wang, Zhengmin Gu, Hang Yu

**Affiliations:** 1 Information Science and Engineering School, Northeastern University, Shenyang, 110004 China; 2 Liaoning Key Laboratory of Intelligent Diagnosis and Safety for Metallurgical Industry, Northeastern University, Shenyang, 110819 China; 3 Shenyang Institute of Computing Technology Co. Ltd., CAS, Shenyang, 110168 Liaoning China; 4 College of Science, Shenyang Jianzhu University, Shenyang, 110168 Liaoning China; 5 The First Hospital of China Medical University, Shenyang, China

**Keywords:** Engineering, Electrical and electronic engineering

## Abstract

In the process of mining belt transportation, various foreign objects may appear, which will have a great impact on the crusher and belt, thus affecting production progress and causing serious safety accidents. Therefore, it is important to detect foreign objects in the early stages of intrusion in mining belt conveyor systems. To solve this problem, the YOLOv4_GECA method is proposed in this paper. Firstly, the GECA attention module is added to establish the YOLOv4_GECA foreign object detection model in the mineral belt to enhance the foreign object feature extraction capability. Secondly, based on this model, the learning rate decay of restart cosine annealing is used to improve the foreign object image detection performance of the model. Finally, we collected belt transport image information from the Pai Shan Lou gold mine site in Shenyang and established a belt foreign body detection dataset. The experimental results show that the average detection accuracy of the YOLOv4_GECA method proposed in this paper is 90.1%, the recall rate is 90.7%, and the average detection time is 30 ms, which meets the requirements for detection accuracy and real-time performance at the mine belt transportation site.

## Introduction

Foreign body detection plays an important role in the ore processing and product quality control industries. Ore conveyor belts can be laden with all kinds of foreign bodies when transporting ore, such as steel bars, iron cables, iron, wood, plastic pipes, etc., which can have a huge impact on crushers, ball mills, and belts. The traditional foreign body detection methods are the manual detection method, ray method, and spectral detection method. The manual detection method is greatly influenced by the mental state of workers and is inefficient. The ray method is more stable but expensive and harmful to the human body^1^. The spectral detection method has a relatively low false detection rate, the disadvantage is that it is susceptible to interference, difficult to maintain equipment, and difficult to detect foreign objects in iron ore for detecting iron. Due to the influence of human factors and external interference, the above foreign body detection techniques are slow, costly, and difficult to maintain, with high leakage rates, making it difficult to promote them universally in mining enterprises.

With the development of deep learning, object detection methods based on convolutional neural networks have been widely used, and foreign body detection methods based on deep learning have become a research hotspot. The existing object detection methods are mainly anchor-based and anchor-freed. Among them, anchor-based mainly has two-stage Faster R-CNN and one-stage YOLO series, SSD, etc. In 2015, Ren^2^ et al. proposed the Faster R-CNN, which improved the speed of the selective search algorithm to extract candidate box regions and became the first end-to-end near real-time deep learning target detector. YOLO (you only look once)^3^ was proposed by Redmon et al. in 2015 as the first single-stage detector in the field of deep learning. The main improvement point of YOLOv2^4^ compared with the previous version is the proposed joint training algorithm, which provides more accurate localization while maintaining the processing speed of YOLO.YOLOv3^5^ features the introduction of FPN for multi-scale prediction, and also uses a better underlying network Darknet-53 and binary cross-entropy loss function, and can achieve a balance between speed and accuracy by changing the network structure of the model.YOLOv4^6^ is a major milestone in the YOLO series, with the introduction of CSPDarknet-53 to extract features, the addition of SPP networks to improve image extraction, and the use of the Mish activation function, these improvements also make YOLOv4 an extremely efficient and powerful object detector. Liu^7^ et al. proposed the SSD algorithm in 2015, which introduces multi-reference and multi-resolution detection techniques, and the network of different layers detects objects with different scales, and the detection effect for small targets is greatly improved. YOLOv5^8^ utilizes adaptive anchor frame computation and a multi-semantic fusion detection mechanism, which enables the fast and effective fusion of rich high-level semantic information with low-level location information to achieve fast detection of objects.YOLOv6^9^ incorporates ideas from recent network design, training strategies, testing techniques, quantification, and optimization methods to build a set of deployable networks of different sizes to accommodate diverse use cases.YOLOv7^10^ designs several trainable Bag-of-Freebies methods that enable real-time target detection to greatly improve detection accuracy without increasing the cost of inference, while effectively reducing about 40% of the parameters and 50% of the computational effort of existing real-time target detectors.

In recent years, a large number of scholars have used deep learning methods for foreign object detection, including convolutional neural network (CNN)-based foreign object detection, recurrent neural network (RNN)-based foreign object detection, and autoencoder-based foreign object detection.These methods have achieved good results in foreign object detection and have better generalization and robustness compared to traditional machine learning algorithms.In 2018, Cao^11^ et al. proposed a novel convolutional neural network (CNN)-based algorithm for detecting foreign object debris (FOD) based on optical imaging sensors. Compared with faster R-CNN and SSD, their proposed algorithm has better results for the detection of foreign object debris for airport pavements.In 2018, Xu^12^ et al. proposed a novel foreign object fragment material recognition method based on migration learning and mainstream deep convolutional neural network (D-CNN) models.In 2019, Rong^13^ et al. applied two different convolutional neural network structures to walnut images for automatically segmenting the images and detecting natural and artificial foreign objects of different sizes, avoiding manual feature extraction and overcoming the clumping phenomenon between walnuts and foreign objects in real images.In 2020, He^14^ et al. constructed a deep learning-based network for plastic bag detection under trains using the SSD network and combining different feature extractors. Among the results, the fastest detection speed was obtained with the combination of SSD and MobileNet.In 2020, Pang^15^ et al. proposed a real-time detection method based on the YOLOv3 algorithm to detect hidden metal weapons on the human body, which was applied to passive millimeter wave (PMMW) images. It not only has high accuracy but also very fast detection speed in terms of detection for targets with small size.In 2022, Chen^16^ et al. combined the YOLOv4 algorithm with an optimized anchor box to achieve efficient detection of foreign objects in belt conveyors and reduce the occurrence of this problem of longitudinal belt tears.In 2022, Qiu^17^ et al. A deep learning-based real-time detection technique for ground radar with added attention mechanism and data augmentation to improve false and missed detection problems in detection.In 2022, Jing^18^ et al. proposed a random forest framework based on optimal pixel vision features and designed pixel vision features (PVF) in order to overcome the complexity of airport pavement image information and the variability of foreign object fragments, which is more advantageous in terms of accuracy and recall of foreign object fragment detection.In 2022, Abramson^19^ et al. created a fully automated foreign object tracking algorithm that used a custom convolutional neural network to achieve 99% accuracy, surpassing other comparable algorithms.

The current research on foreign body detection in mining belts focuses on traditional object detection methods, with low recognition accuracy, slow identification speed, and easy to occur the situation of leakage and misjudgment. At the same time, there are small objects foreign bodies, foreign bodies partially obscured and foreign bodies difficult to identify due to dust interference during mineral belt transportation. To solve the above problems, this paper proposes a YOLOv4_GECA-based method to detect foreign bodies on the ore transmission belt. Our contribution can be summarized as follows.

1. In response to the lack of a standard and open image database in the field of mining belt foreign body detection, we collected belt transport image information from the Pai Shan Lou gold mine site in Shenyang, created a mining belt foreign body detection dataset, and expanded it, with the main foreign body types including steel bars, steel cables, iron, wood, and plastic pipes.
2. The GECA attention mechanism is proposed and the YOLOv4_GECA model is built for the characteristics of partial occlusion of foreign bodies and dust interference in images in the actual production process.
3. The learning rate decay method in the model training process is improved to optimize the training process with the restart cosine annealing learning rate decay method. Based on the YOLOv4_GECA model, the YOLOv4_GECA_SGDR network is constructed to further improve the performance without increasing the model burden.

## Related work

### YOLOv4 network structure

**Figure 1:**
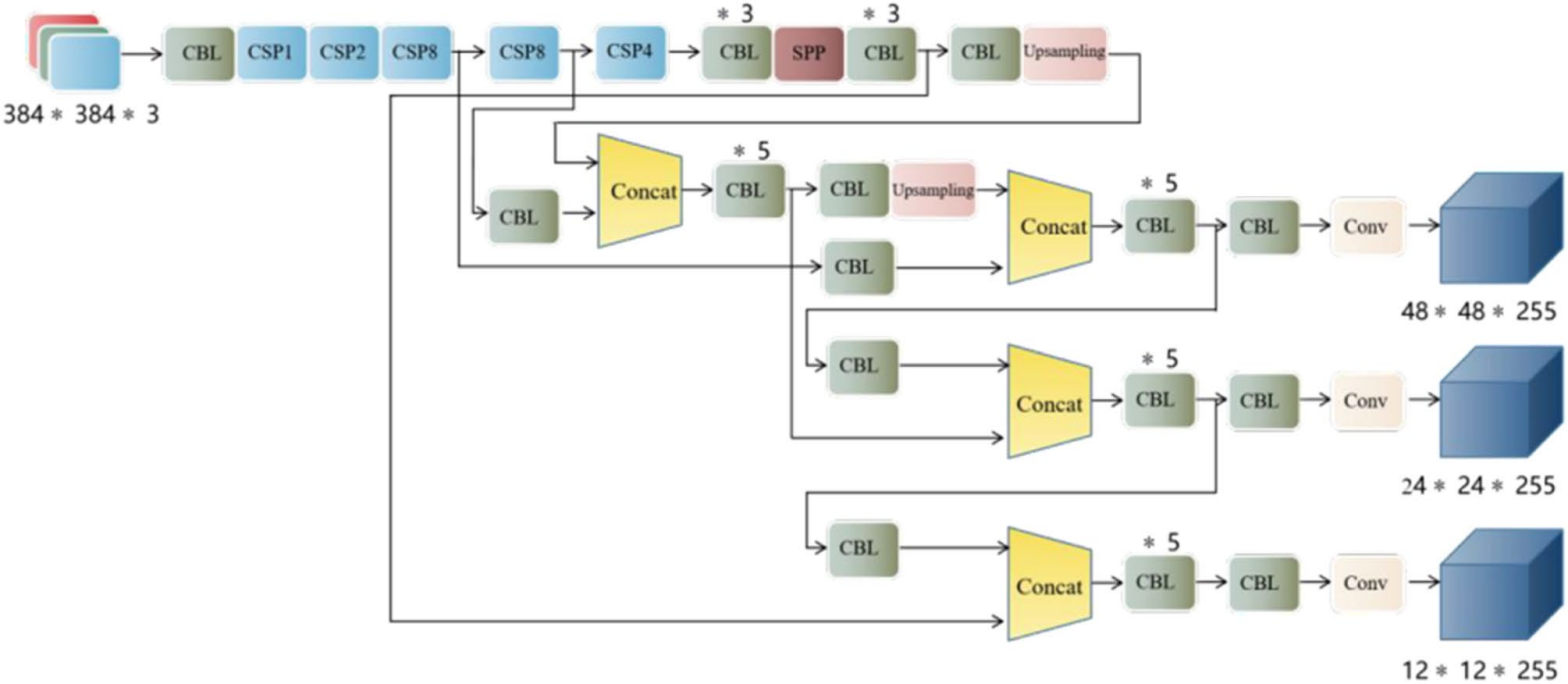
The structure of the YOLOV4 model.

The YOLOv4 model is improved based on the YOLOv3 model^20^, incorporating some excellent detection techniques that have emerged in the field of deep learning in recent years, making the model's detection performance for small objects improved, and the schematic diagram of the model structure is shown in Fig. 1. In terms of the overall network structure, the YOLOv4 model can be divided into four parts: input, backbone network, neck, and head. The YOLOv4 model uses Mosaic data augmentation on the input side during training by performing some operations of flipping and scaling the four images separately and then stitching the four images together to get a new image^21^. The YOLOv4 model backbone includes the use of the CSPDarknet53 network and the Mish activation function. The object detection network uses a spatial pyramidal pooling structure and a PANet structure in the middle of the CSPDarknet53 and output layers. PAN passes the deep semantic information to the shallow layer to complement the shallow semantic information, thus obtaining high-resolution and strong semantic features, which have very impressive performance in areas such as small object detection and instance segmentation. On Prediction, the anchor frame mechanism of the output layer is the same as YOLOv3, and the main improvements are the loss function CIOU_Loss during training and the non-maximum suppression of the prediction frame screening into DIOU_nms.

### CSPX module

**Figure 2:**
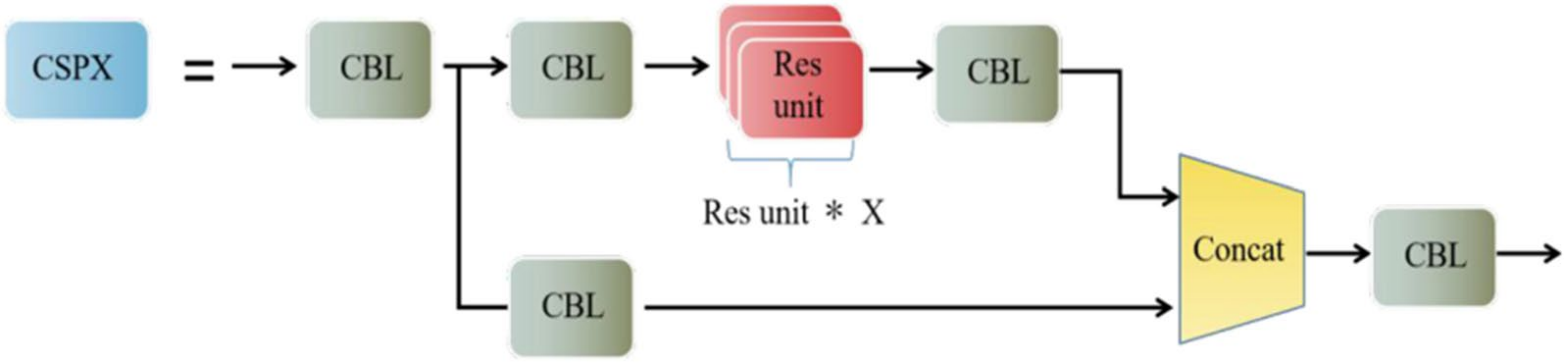
Structural diagram of the CSPX module.

**Figure 3:**
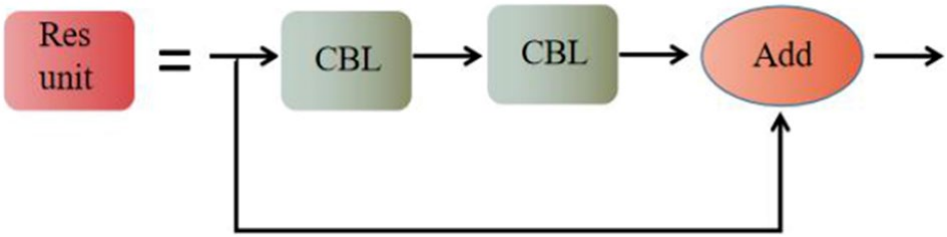
Structural diagram of the Res unit module.

**Figure 4:**
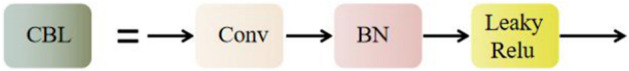
Structural diagram of the CBL convolution module.

YOLOv4's CSPDarknet53 backbone network architecture uses many CSPX modules for stacking. The CSPX module borrows the structure of the cross-stage partial network^22^ (CSPNet) with tensor stitching by convolutional layers and X Res unit modules, see Fig. 2. In the object detection problem, the use of CSPNet as the backbone network can bring some performance improvement to the network model, enhance the ability of convolutional neural networks to extract features from images, and improve computational efficiency. Figure 3 shows the structure of the residual module (Res unit), which is divided into a direct mapping part and a residual part, where the residual part contains two convolution operations. The residual part is first convolved and then tensor-summed with the direct mapping part. Figure 4 illustrates the CBL convolutional module, which consists of a Convolutional layer, a Batch Normalization layer, and a Leaky Relu activation layer.

### ECA attention mechanism

**Figure 5:**
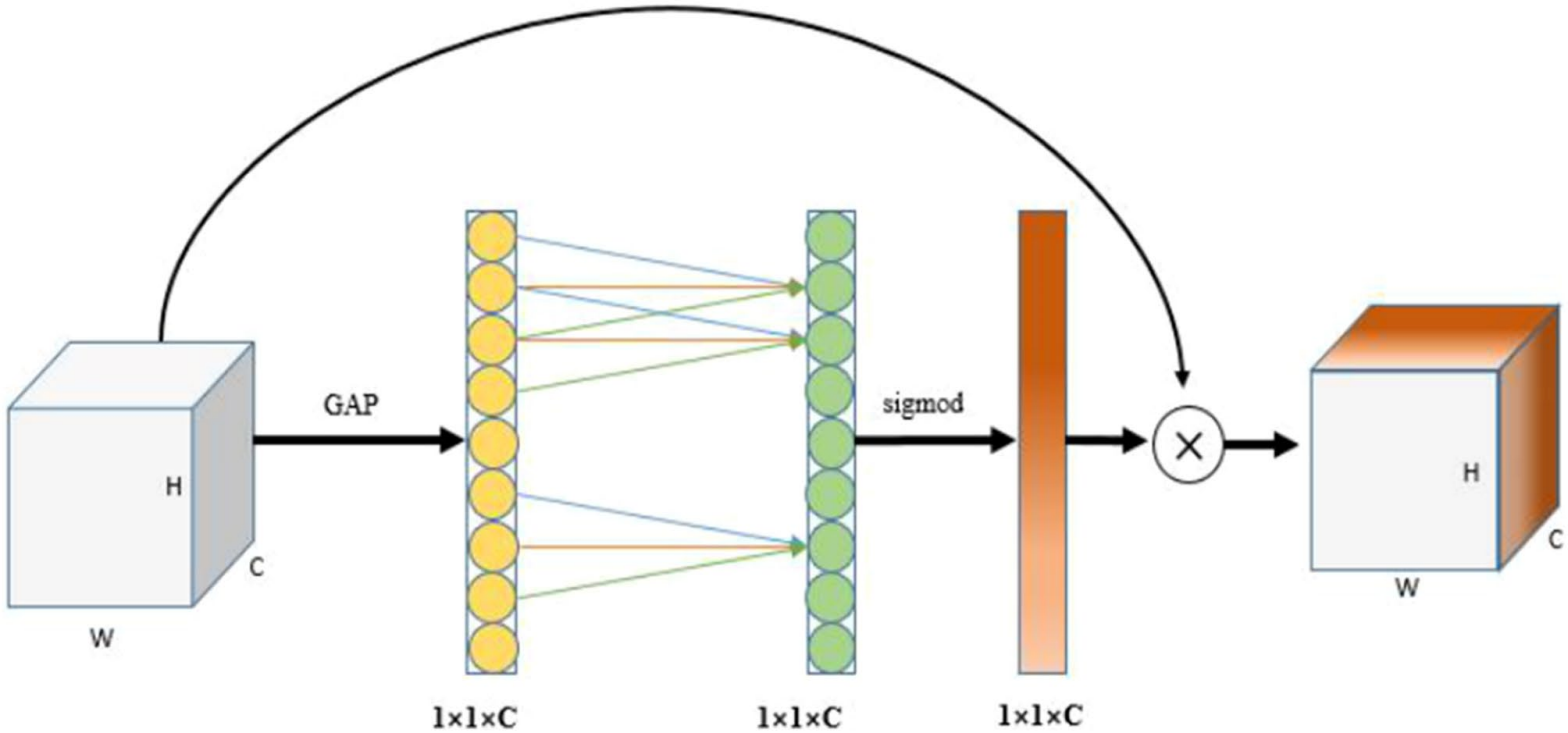
Structural diagram of the ECA module.

The ECA attentional mechanism module improves on the SE module^23^ by eliminating the dimensionality reduction operation and using two fully connected layers of the same dimension more helpful for learning effective channel attention. However, using two fully connected layers of the same dimension will lead to an excessive amount of parameters, so the approach of using k adjacent features to obtain local cross-channel interaction information has only k number of parameters and can get the same performance and is more efficient. The ECA attentional mechanism module is calculated as:

1 $$\omega = \sigma \left( {C1D_{k} \left( y \right)} \right)$$

where $C1D$ represents the one-dimensional convolution operation performed on the input features and represents the Sigmoid function. The ECA module does not use the full connection layer in the SE module. It directly learns the features after the global average pooling through a one-dimensional convolution that can share weights, as shown in Fig. 5. The local cross-channel interaction information is obtained by each channel and its k adjacent features, i.e., the information interaction between channels is achieved by one-dimensional convolution with a convolution kernel of size k. The nonlinear relationship between each channel is learned and the weights of different channels are also obtained. One-dimensional convolution involves the hyperparameter k, the size of the convolution kernel, which indicates the coverage of local cross-channel interactions, i.e., how many adjacent features in the vicinity of that channel feature are jointly involved in attention prediction.

### Cosine annealing learning rate mechanism

**Figure 6:**
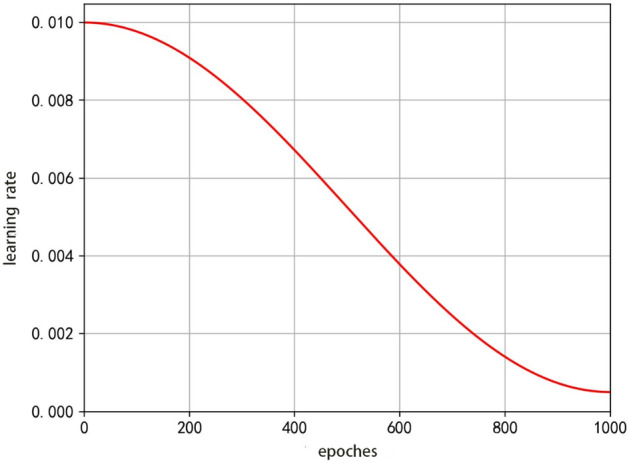
Cosine annealing learning rate descent.

During the optimization of the stochastic gradient descent algorithm, as the loss value approaches the global minimum, the current learning rate is large for the current weights and loss function, and at this point, if the learning rate is reduced the model can be brought closer to the global minimum. Cosine annealing reduces the learning rate by the cosine function, as shown in Fig. 6, where the horizontal coordinate is the number of training rounds and the vertical coordinate is the learning rate. The decay of the learning rate from 0.01 to 0.005 with increasing training rounds is illustrated in Fig. 6, where its cosine value first decreases slowly with increasing rounds, then accelerates and decreases slowly again. This is used in the training process of the YOLOv4 model, where the learning rate decays gradually as training proceeds, with the loss function first decreasing substantially early in the training, and varying in a small range around the local optimum or global optimum later.

The cosine annealing learning rate decay method uses the cosine function, so the decay process conforms to the form of the cosine function, whose formula is shown in Eq. (2).

2 $$\eta_{{\text{t}}} = \eta_{\min }^{{}} + \frac{1}{2}\left( {\eta_{\max }^{{}} - \eta_{\min }^{{}} } \right)\left( {1 + \cos \left( {\frac{{T_{cur} }}{{T_{{}} }}\pi } \right)} \right)$$

where ${\eta }_{max}$ and ${\eta }_{min}$ are the maximum and minimum values of the learning rate, respectively, ${T}_{\text{cur}}$ is the number of rounds trained so far, and $T$ is the total number of training rounds.

Stochastic gradient descent optimization algorithms often encounter the problem of falling into local minima during the training process. The cosine annealing learning rate decay approach will have a smaller learning rate in the later stages of training and may fall into local minima that are difficult to jump out of, resulting in a descent algorithm that cannot optimize to a global minimum. If the learning rate is increased at a time when the learning rate is small later in the training of the stochastic descent algorithm, then it is possible to make the optimization process jump out of the local minima and find the path to the global minimum, called restarting the cosine annealing learning rate method. The formula for the restart cosine annealing learning rate is given in Eq. (3).

3 $$\eta_{{\text{t}}} = \eta_{\min }^{t} + \frac{1}{2}\left( {\eta_{\max }^{i} - \eta_{\min }^{i} } \right)\left( {1 + \cos \left( {\frac{{T_{cur} }}{{T_{i} }}\pi } \right)} \right)$$

**Figure 7:**
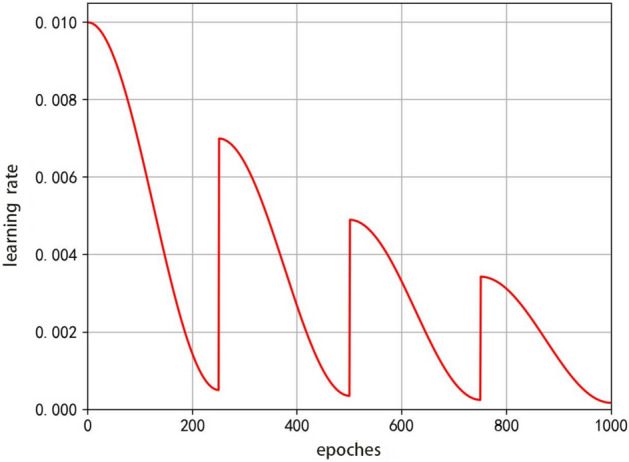
Restart cosine annealing learning rate descent.

In the formula, the $i$ denotes the first few times the restart learning rate. The ${\eta }_{max}^{i}$ and ${\eta }_{min}^{i}$ denote the maximum and minimum values of the learning rate in the ith restart, respectively. ${T}_{i}$ denotes the total number of rounds of the training process in the ith restart and ${T}_{\text{cur}}$ denotes how many rounds are currently executed and will be updated in each restart. The maximum and minimum values in the restart learning rate are set, and each restart learning rate undergoes a cosine annealing decay, with the learning rate decaying from the maximum value to the minimum value. Figure 7 shows a simulation of the learning rate for three restart cosine annealing, where the learning rate is restarted in the 50th, 100th, and 150th rounds of the training process, and the maximum and minimum value of the learning rate for each restart is seventy percent of the previous value.

## Build the YOLOv4_GECA model

### Build GECA to improve the attention mechanism

**Figure 8:**
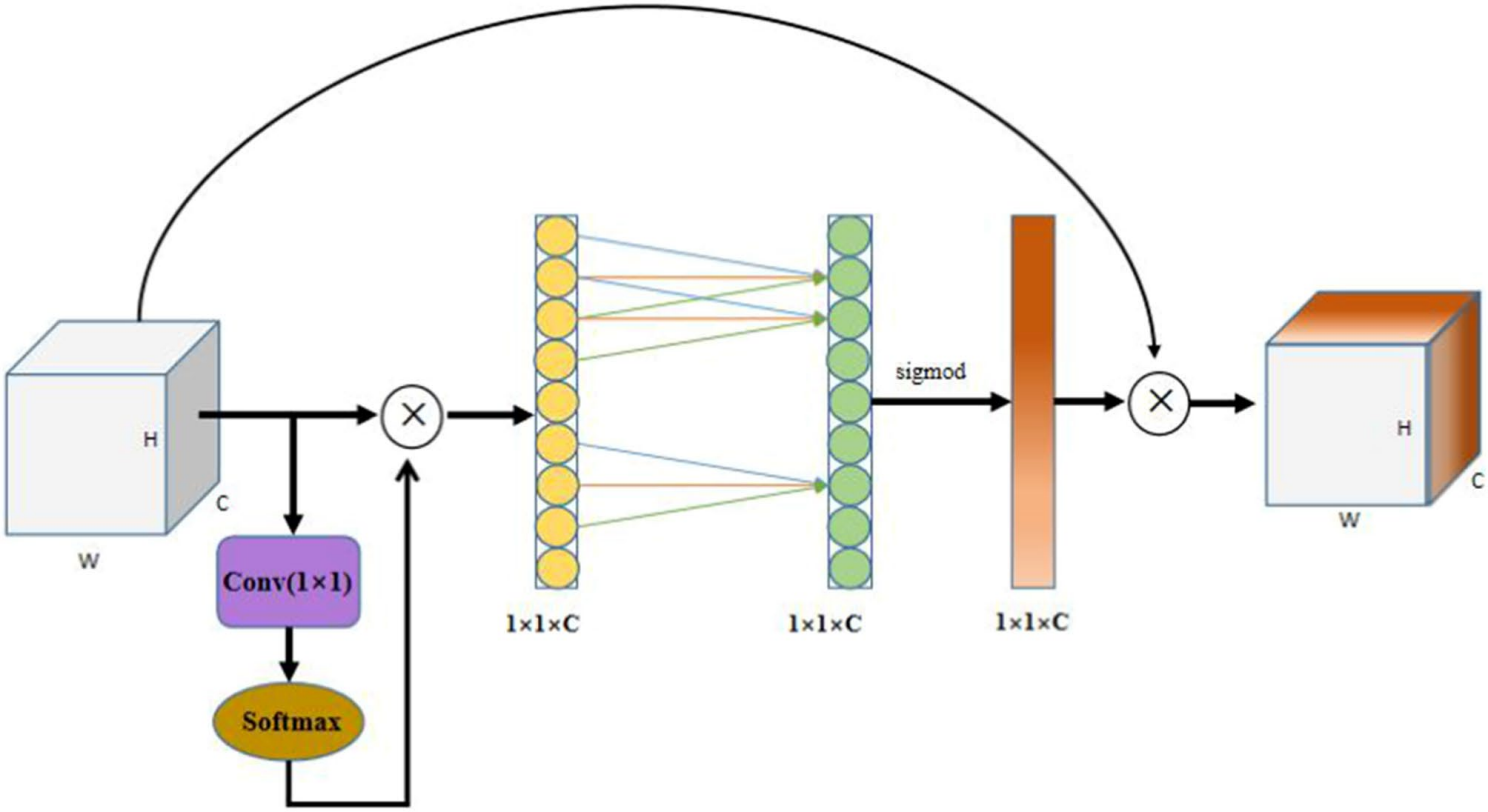
Structural diagram of the GECA module.

To make full use of the global contextual information and capture long-range dependencies for the actual production site where there are small objects, some foreign objects exist to obscure and dust may be generated, this paper proposes the GECA attention mechanism to improve the ECA attention mechanism. The global average pooling operation in the ECA attention mechanism only operates by averaging each channel feature map separately and uses this average to represent the channel feature map information without making full use of the global information. In this paper, the global averaging pooling operation in ECA is improved by not using global averaging pooling, but by using a one-dimensional convolution operation to obtain the dependencies on the pixel locations of each channel feature map to obtain the output vector, which is later operated by the Softmax function to obtain the probability value vector, which is multiplied with the original input feature map. This improvement can make full use of the full-text information of the feature map to obtain the connection between the pixel points of each channel, and the obtained probability value vector makes the key information on the feature map more prominent, which is more convenient for the subsequent learning of the attention mechanism of the channel, and enhances the feature extraction ability of partially occluded foreign objects and foreign objects on dusty images as well. The built GECA module is shown in Fig. 8.

### Build Res_GECA to improve the residual module

**Figure 9:**
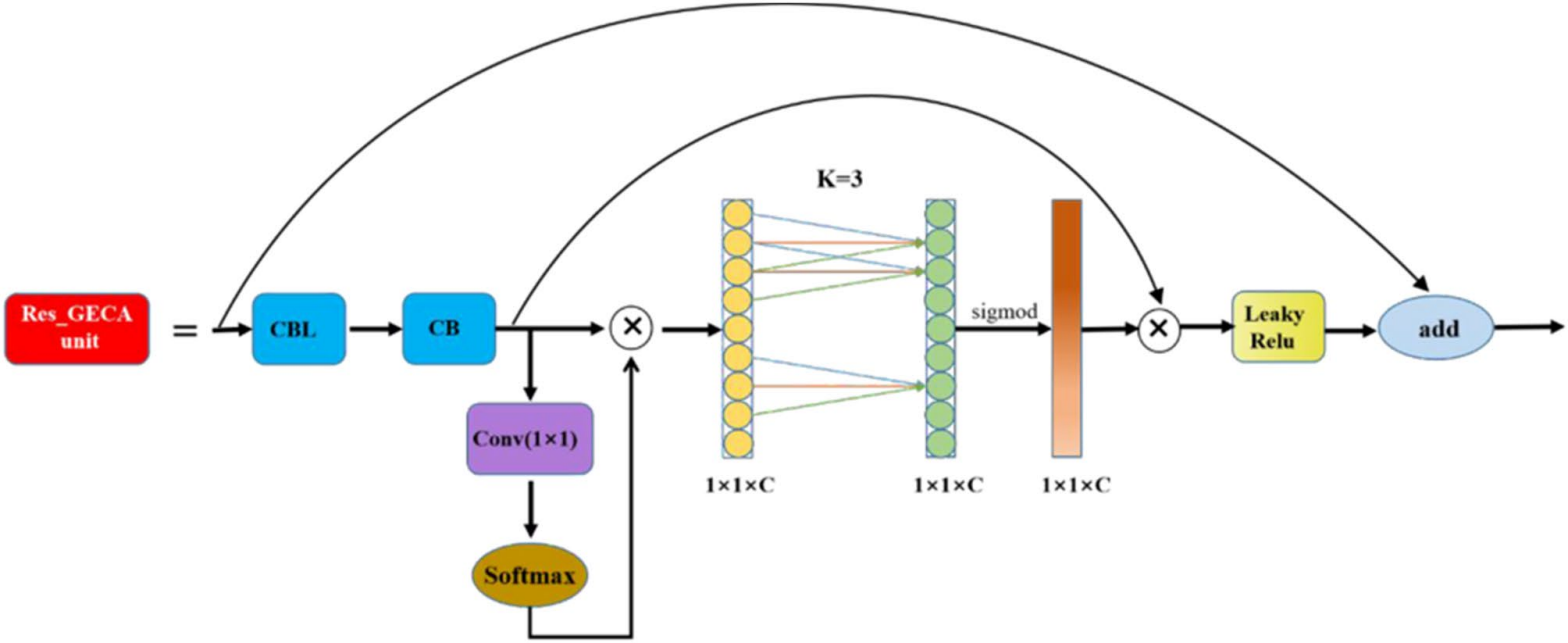
Structural diagram of the Res _GECA unit module.

The CSPX module in the CSPDarknet53 backbone network contains several residual blocks Res units that mitigate the gradient disappearance and gradient explosion problems, allowing the network structure to be built deeper and more capable of extracting features from images. In this paper, we propose to construct the Res_GECA unit module by placing the attention mechanism module GECA behind the convolutional layer of the residual part in the residual block of the Res unit, expecting to learn the channel attention of the convolutional block more effectively and improve the feature extraction ability of the backbone network with the introduction of very small model parameters. The constructed Res_GECA unit module is shown in Fig. 9.

### Build YOLOv4_GECA model

**Figure 10:**
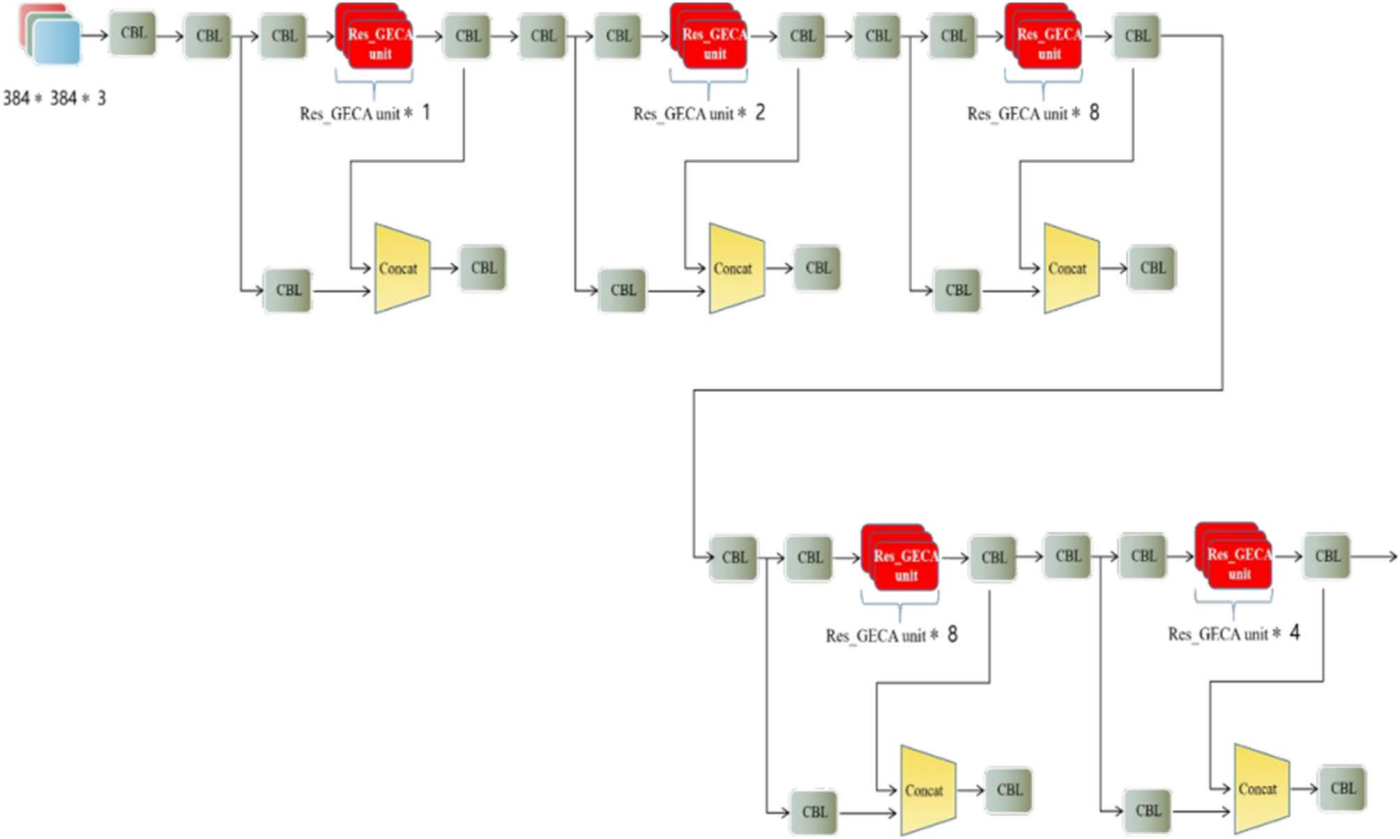
The backbone network structure diagram of the YOLOV4_GECA model.

There are 23 residual connection modules in the five CSPX modules of the YOLOv4 backbone network, and the original residual connection module is replaced by the Res_GECA residual module with the GECA attention mechanism proposed in this paper, see Fig. 10. This builds the YOLOv4_GECA model, which constructs a backbone network with an attention mechanism to increase the learning ability of the model on the importance of the feature map channel level at the cost of increasing fewer model parameters, fully exploiting the feature map information, enhancing the useful feature map information, and enabling improved detection of hard-to-identify foreign bodies.

## Experiments and analysis of results

### Mining belt foreign body data set

Currently, there is no standard and open image database in the field of mining belt foreign body detection. We collected belt transport image information from the Pai Shan Lou gold mine site in Shenyang, created a dataset, and expanded the dataset using data enhancement. The image information in the dataset can be seen in the Supplementary Information. Data enhancement is an artificial expansion of a limited data set to produce more equivalent data. Commonly used image data enhancement methods include image color dithering, flip and rotate transformations, random trimming, pan and contrast transformations, Mosaic data enhancement, etc. In this paper, image rotation and flip transforms, and image color dithering are used to simulate the possible poses and field conditions of foreign objects on the ore conveyor belt in a random way.

This paper uses LabelImg, a labeling software for target detection tasks, which allows the target to be selected using a rectangular box and saved as an XML file. To create a sample set of foreign object images of the mining belt, the LabelImg dataset labeling software was used to manually map the foreign object targets on the conveyor belt.To mark a single foreign object, click the rectangular box button on the software, hold down the left mouse button in the upper left corner of the foreign object, slide the mouse down to the lower right corner of the foreign object, let the rectangular box completely wrap around it, then fill in the foreign object category and save it. When there are multiple foreign objects in the diagram, mark each foreign object separately and fill in the category it belongs to. The labeling is saved in VOC format. A total of 1291 images containing foreign objects were selected for the final total data set, which was divided into 5 foreign object categories, including steel, plastic pipe, wood, steel cable, and iron, with a balanced number of images in each category.The training set and test set are taken in the ratio of 8:2. Mosaic data augmentation is used during training to enrich the data set and make the network more robust.

### Training environment

The computer used for the training of the mining belt foreign object detection model in this paper uses an Intel Core i5-9400F 2.90 GHz processor, an NVIDIA GTX 1660 graphics card, 16 GB of RAM, and a 500 GB Western Digital hard drive. In terms of software, the computer operating system is Windows 10 64-bit. The mining belt foreign body detection model is built using the PyTorch deep learning framework, which is a widely adopted, simple, and efficient open-source framework. Opencv and PIL image processing libraries are used to process the images, and libraries such as Matplotlib are used to draw the images. For the GPU, use Cudnn 7.6.5 and Cuda 10.2 to configure the graphics card.

### Training methods

The YOLOv4 model uses CSPDarknet53 as the backbone architecture and is pre-trained on the ImageNet dataset to initialize some of the convolutional layers. In the experiment, the default hyperparameters are as follows: the total number of training rounds for the mining belt foreign body detection model is 1000 rounds; the size of each batch training image is set to 4; the initial value of the learning rate is set to 0.01, which will slowly decay during the training process, and the final decay learning rate is 0.0005; the momentum and weight decay are set to 0.937 and 0.005, respectively; the size of the input image is 3843´84. where the one-dimensional convolutional kernel size in the YOLOv4_GECA model is set to 3, 5, and 7 for experiments to test the performance impact of this parameter on model improvement. In terms of selecting the activation function, we use the Leaky Relu activation function because the graphics card capacity required for Mish activation is too large. The mining belt foreign body detection model uses a stochastic gradient descent optimization algorithm, which first uses a cosine annealing decay learning rate scheduling strategy to find a suitable improved model, and then trains the model using a restarting cosine annealing learning rate decay strategy to test whether different learning rate decay strategies affect the model. For testing, we obtained region-level confidence vectors by scores. The results are post-processed by bounding box voting and Non-Maximum Suppression (NMS) using a 0.5 IOU threshold.

### Evaluation indicators

In the process of conveyor belt transport ore site detection of foreign bodies, the occurrence of false and missed foreign object detection and detection speed is not up to standard on the inspection work has a great impact, so the verification indicators of these situations as the main performance indicators of the detection system of this study. Therefore, Mean Average Precision (mAP), Recall, and Fps (frames per second) are used as model evaluation metrics in this study. Where Mean Average Precision (mAP) is the average of AP values taken over all categories. mAP is the average accuracy, which is the average of all accuracies obtained over all possible values of Recall. The recall is used to evaluate the detection coverage of the detector for all targets to be detected. Fps refers to the number of images that can be processed in each second. They are calculated as follows.

4 $${\text{Precision}} = {\text{TP}}/\left( {{\text{TP}} + {\text{FP}}} \right)$$

5 $${\text{Recall}} = {\text{TP}}/\left( {{\text{TP}} + {\text{FN}}} \right)$$

6 $${\text{AP}} = \mathop \smallint \limits_{0}^{1} {\text{P}}\left( {\text{R}} \right){\text{dR}}$$

7 $${\text{mAP}} = \frac{1}{{\text{Q}}}\mathop \sum \limits_{{{\text{q}}\epsilon {\text{Q}}}} {\text{AP}}\left( {\text{q}} \right)$$

In the formula,TP is the number of positive samples predicted to be positive classes; FP is the number of negative samples predicted to be positive classes; FN is the number of positive samples predicted to be negative classes; TN is the number of negative samples predicted to be negative classes.

### Experiment and analysis

This paper compares the detection effects of different advanced object detection models on mining belt foreign body detection dataset, including five network models, YOLOv3, YOLOv3-spp, Faster RCNN, YOLOv4, and YOLOv5, and the YOLOv4 object detection model is selected as the base network model for comprehensive ore transportation site conditions. The performance of the improved models YOLOv4_ECA, YOLOv4_GECA, and YOLOv4 models are also compared with each other, as well as the effect of the one-dimensional convolutional kernel size in the YOLOv4_GECA model on the model performance. In this paper, we improve the learning rate decay strategy in the training of the mining belt foreign body detection model and change the learning rate decay strategy of YOLOv4 to restart cosine annealing learning rate decay and conduct experimental comparison and analysis.

### Performance comparison of the base model on the mining belt foreign body dataset

**Figure 11:**
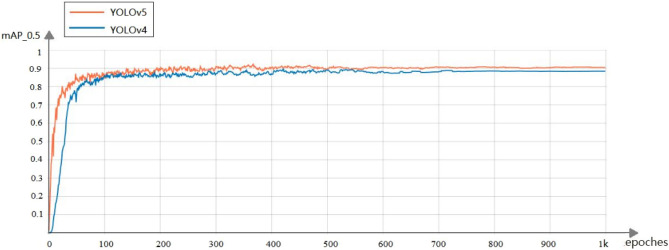
mAP@0.5 indicator changes during YOLOv4 and YOLOv5 model training.

**Figure 12:**
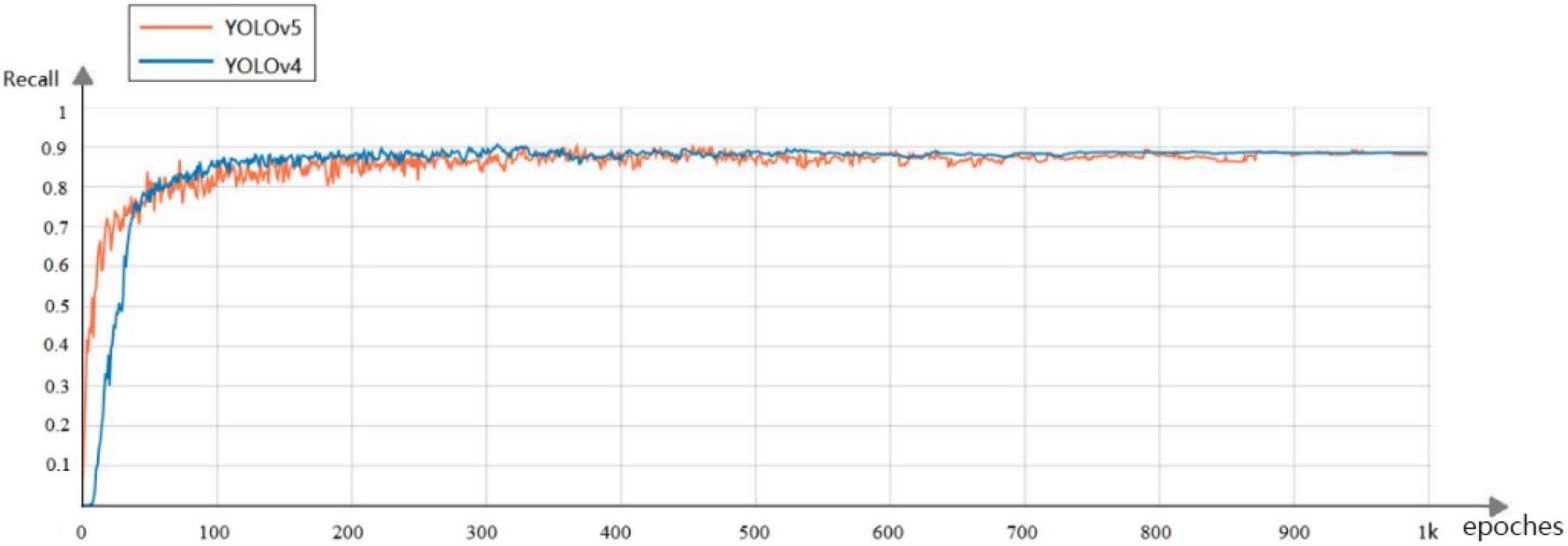
Recall indicator changes during YOLOv4 and YOLOv5 model training.

To demonstrate and evaluate the performance of our proposed method, the performance is compared with other advanced detectors. The five network models, YOLOv3, YOLOv3-spp, Faster RCNN, YOLOv4, and YOLOv5, were trained for 1000 rounds on the mining belt foreign body dataset and tested on the test set. Figure 11 shows the trends of the mAP metrics of the YOLOv4 model and YOLOv5 model during the training process. After 300 rounds of training, the metrics leveled off and varied around the optimal values. Among them, the training effect of the YOLOv5 model is better than that of the YOLOv4 model. Figure 12 shows the trend of Recall metrics during the training of the YOLOv4 model and YOLOv5 model, and after 300 rounds of training, the metrics leveled off and varied around the optimal values. Among them, the training effect of the YOLOv4 model is better than the training effect of the YOLOv5 model.

In this study, the model was trained on a homemade mining belt foreign body dataset, and firstly, the five network models, YOLOv3, YOLOv3-spp, Faster RCNN, YOLOv4, and YOLOv5, which are more popularly used in detection tasks, were trained on the divided training set dataset for 1000 rounds respectively, and then tested on the test set dataset. as shown in Table 1.

**Table 1:** Comparison of detection performance among YOLOv3, YOLOv3-spp, Faster RCNN, YOLOv4, YOLOv5.

Model	mAP@0.5	Recall	Fps
YOLOv3	0.885	0.879	40.39
YOLOv3-spp	0.884	0.873	39.74
Faster RCNN	0.905	0.881	22.36
YOLOv4	0.885	0.888	35.77
YOLOv5	0.907	0.867	118.14

As can be seen in Table 1, the YOLOv5 model has the highest detection accuracy with an mAP of 0.907 for the mining belt foreign body detection task, and the other models have a closer mAP value of about 0.885. However, the YOLOv5 model has a lower Recall of 0.867 and the YOLOv4 model has the highest Recall of 0.888, which is 2.1% higher than the YOLOv5 model. For this testing task, missed detections are more serious compared to false positives, so the Recall is more important. In summary, the YOLOv4 model performs better than the YOLOv3, YOLOv3-spp, and YOLOv5 models in terms of accuracy and Recall, with an mAP of 0.885 and a Recall of 0.888, and its Fps can meet the actual needs of production sites, so the YOLOv4 model is selected as the base model for improvement.

**Figure 13:**
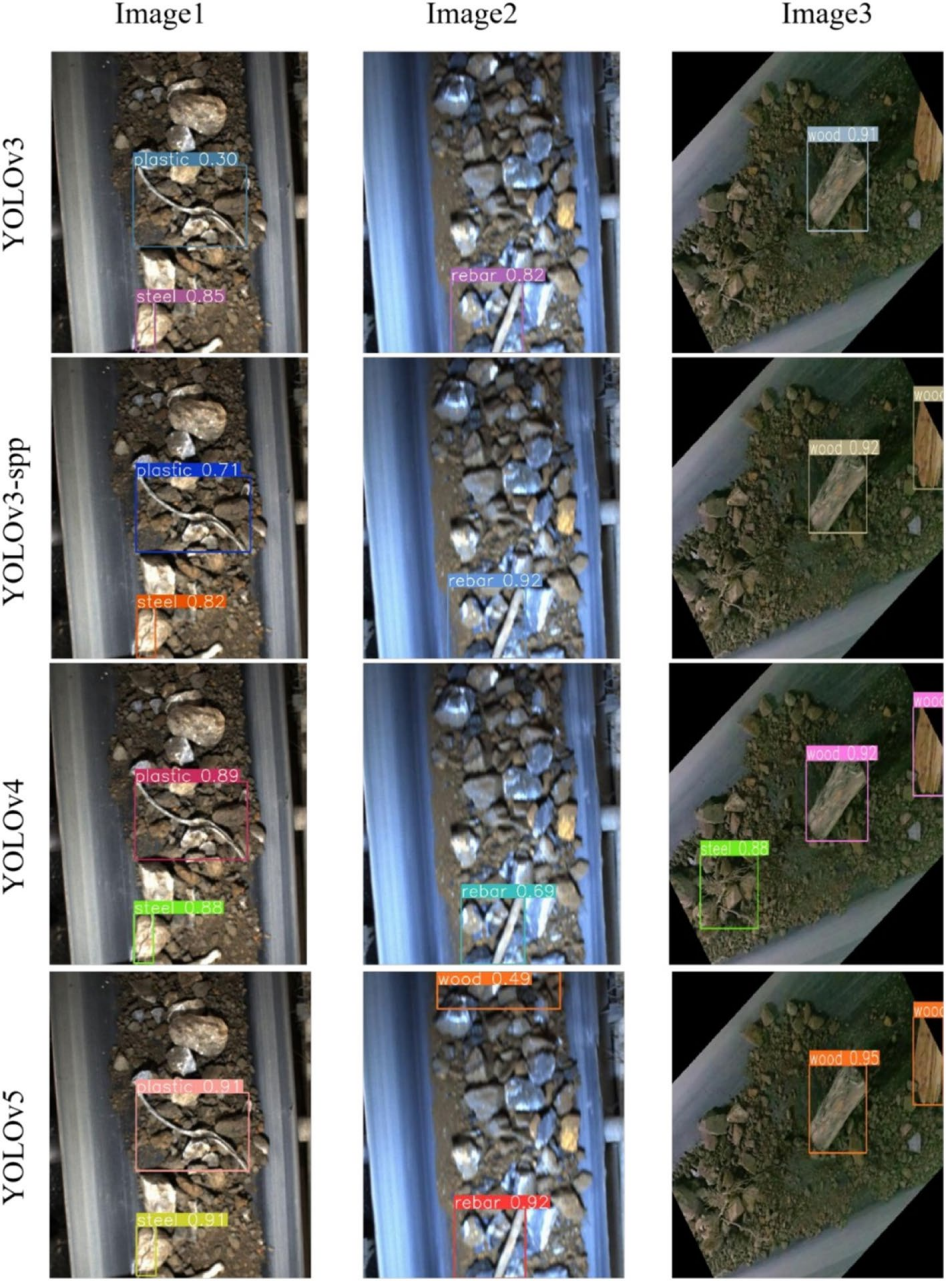
Comparison of the detection effect of the four models.

A comparison of the partial prediction results of the four models YOLOv3, YOLOv3-spp, YOLOv4, and YOLOv5 is shown in Fig. 13. From left to right, three images containing foreign bodies, including wood, rebar, and plastic pipe, and steel cable, are shown in order from top to bottom, showing the detection effects of the four object detection models on the foreign body images.

In the detection of Image1 images, the YOLOv4 model and the YOLOv5 model predicted the categories with higher confidence. In the detection of Image2 images, the YOLOv5 model incorrectly identifies the ore as a wood foreign body relative to the other three models in a case of misclassification. In the detection of the Image3 images, all three models except the YOLOv4 model had missed detection, the YOLOv3 model missed a wood foreign body and a steel cable foreign body, and the YOLOv3-spp model and the YOLOv5 model missed a steel cable foreign body. In the actual production site, both misjudgment and missed foreign body detection can cause trouble to the ore transfer processing process. Missed detection leads to foreign body intrusion into the feed opening, crusher and ball mill, and other equipment causing equipment damage, and misjudgment can increase workers' labor intensity. From the comprehensive effect of model prediction, the YOLOv4 model is more suitable for the mining belt foreign body detection task in terms of performance, so YOLOv4 is chosen as the base model for this paper for model improvement.

### Experimental comparison between the YOLOv4_GECA model and the YOLOv4 model

**Figure 14:**
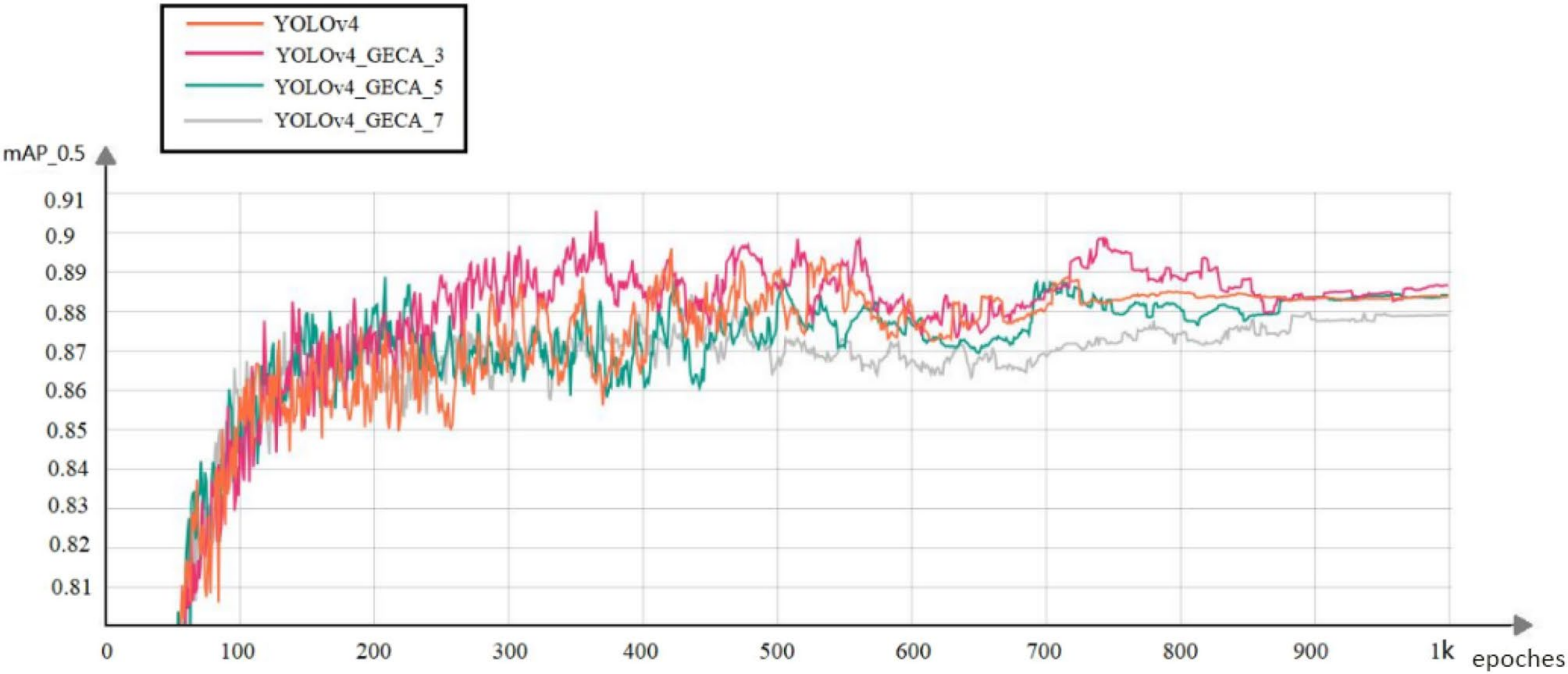
mAP@0.5 indicator changes during YOLOv4_GECA model training.

The improved model YOLOv4_GECA is trained on the mining belt foreign body dataset, and the hyperparameters k involved in the one-dimensional convolution in the Res_GECA residual module are adjusted and set to 3, 5, and 7 respectively, and then trained for 1000 rounds respectively,Several models in the training process mAP@0.5 See Fig. 14 for comparison of indicator change trend. Four curves, YOLOv4, YOLOv4_GECA_3, YOLOv4_GECA_5, and YOLOv4_GECA_7, are shown in Fig. 14, where YOLOv4_GECA_3 indicate that the one-dimensional convolutional kernel hyperparameter k of the GECA attention mechanism in the YOLOv4_GECA model is 3, and so on. From the figure, it can be seen that the mAP@0.5 of the YOLOv4_GECA model with three different k parameters reached their highest point at roughly 400 rounds during the training process of the model, and fluctuated around the optimal value after that. In comparison, the mAP@0.5 of the YOLOv4_GECA model is higher than that of the YOLOv4 model during the training process.

The model was tested on the test set after training, and the test results are shown in Table 2 The detection performance results of the YOLOv4 model and the improved model YOLOv4_GECA with different hyperparameters k are shown in Table 2. It can be seen that the mAP@0.5 of YOLOv4_GECA improves a lot compared to YOLOv4 for the mining belt foreign body detection task, and the Recall of YOLOv4_GECA_3 and YOLOv4_GECA_5 also improves compared to YOLOv4, and the number of parameters of the improved model increases very little. The YOLOv4_GECA_7 model performs better in the mAP@0.5 index, but its Recall decreases. the YOLOv4_GECA model decreases in the Fps index compared to the YOLOv4 model, detecting 5 fewer images per second. The YOLOv4_GECA_3 model works best in terms of all indexes, and it improves the mAP@0.5 and the Recall of the mining belt foreign body detection task by 1% and 0.6%, respectively, and the additional number of parameters resulting from the addition of the GECA attention mechanism is very small. From the experimental results, combining the GECA attention mechanism proposed in this paper with CSPDarknet53, the backbone network of the YOLOv4 model, can improve the foreign body detection performance. Although the YOLOv4_GECA_3 model has reduced in Fps index, detecting 30 foreign object images per second, it can still meet the demand of foreign object detection task of mining belt from the practical situation and can be applied in the actual production process.

**Table 2:** Comparison of detection performance among YOLOv3, YOLOv3-spp, Faster RCNN, YOLOv4, YOLOv5.

Model	mAP@0.5	Recall	Parameters(× 10^6^)	Fps
YOLOv4	0.885	0.888	63.9592	35.77
YOLOv4_GECA_3	0.895	0.894	63.9593	30.07
YOLOv4_GECA_5	0.891	0.892	63.9594	30.18
YOLOv4_GECA_7	0.890	0.886	63.9594	30.19

### Comparison of restart cosine annealing learning rate decay experiments

**Figure 15:**
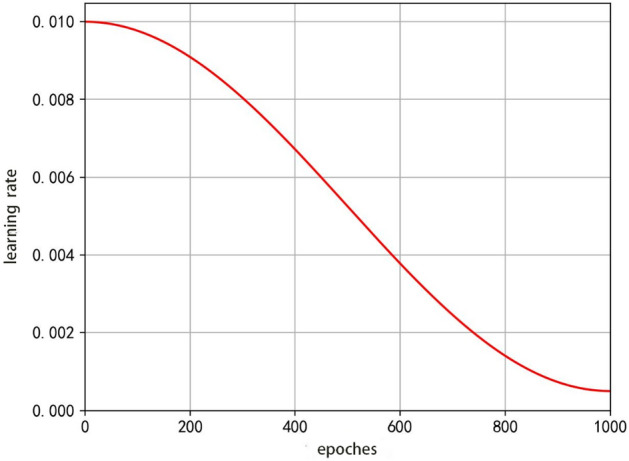
The decay process of the learning rate of the YOLOV4 model.

A restart cosine annealing learning rate mechanism is used in the training process of the improved foreign body detection model YOLOv4_GECA_3 on the mining belt foreign body dataset. The YOLOv4 model is trained with the cosine annealing learning rate mechanism, and its learning rate decay process is shown in Fig. 15. The initial learning rate value is 0.01, which decays slowly at the beginning of the training phase and decreases rapidly as the training proceeds, and decreases at the final stage of the model training, with a final learning rate of 0.0005.

**Figure 16:**
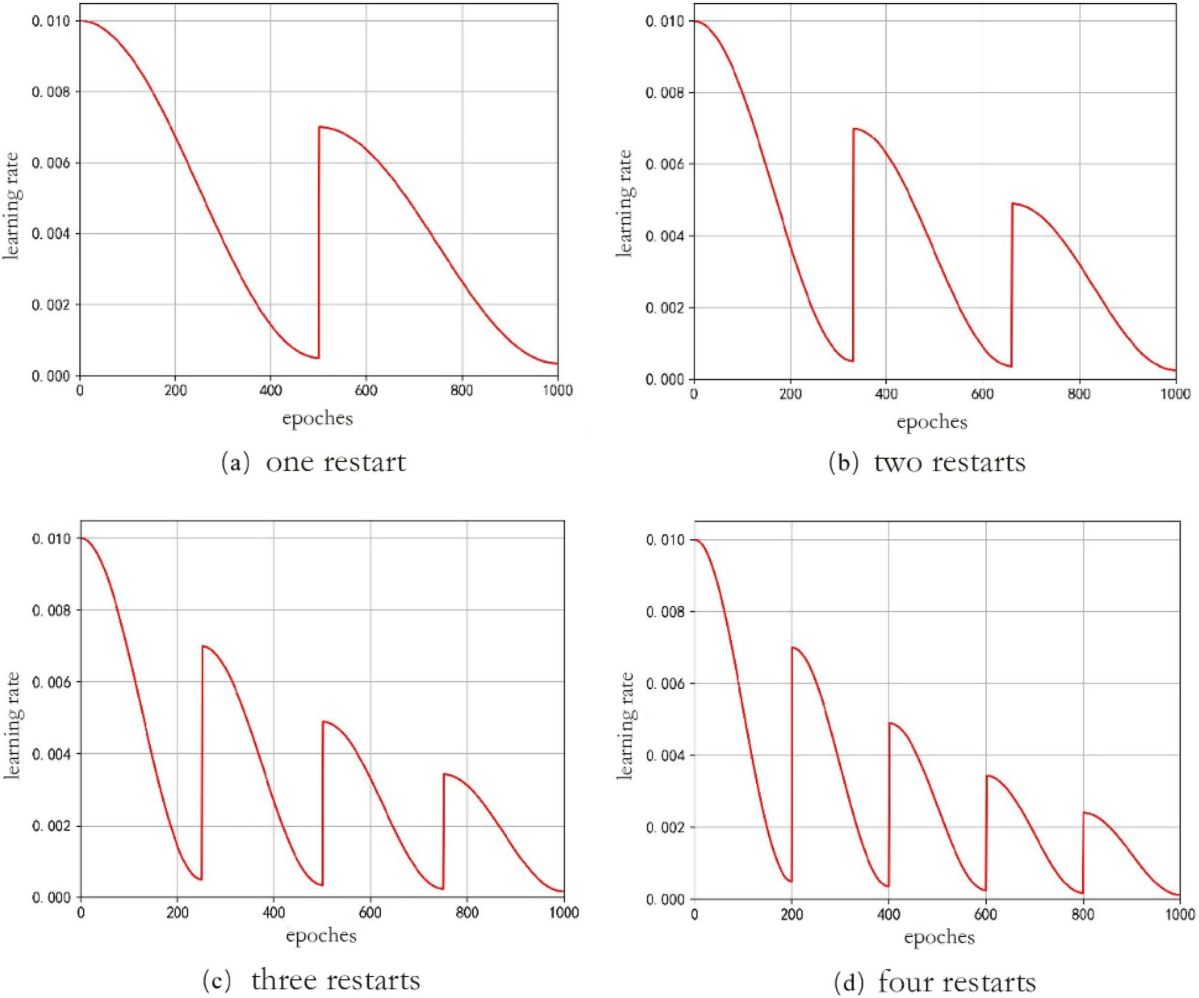
Set different restart cosine annealing learning rates.

Since the learning rate decay of cosine annealing is small in the late stage of model training and may fall into local minima, this paper uses a restart cosine annealing learning rate mechanism to increase the learning rate when the learning rate is small in the late stage of stochastic gradient descent algorithm training, so that the optimization process jumps out of local minima and finds the path to the global minimum. In this paper, the learning rate of one restart, the learning rate of two restarts, the learning rate of three restarts, and the learning rate of four restarts were tested, as shown in Fig. 16. In Fig. 16, the graphs of the learning rate of cosine annealing for a different number of restarts are shown, from left to right from top to bottom, primary restart, secondary restart, tertiary restart, and quadruple restart, with an initial learning rate of 0.01 and a final learning rate of 0.0005. The restart points are located at the points where the training rounds are evenly divided, such as four restart learning rates, whose restart points are located when the model is trained to the 200th, 400th, 600th and 800th rounds, respectively. The starting value of each restart of the learning rate is the maximum value of the previous learning rate multiplied by a scaling factor, which is used in this paper as 0.7.

**Figure 17:**
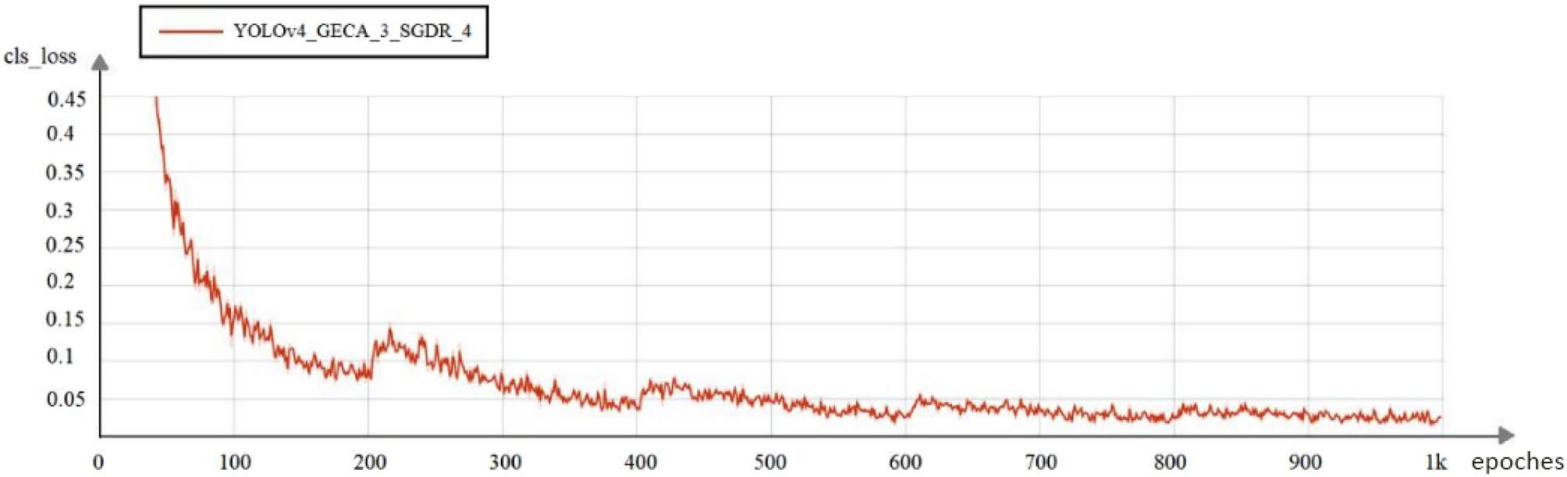
Classification loss changes during YOLOv4_GECA_3_SGDR_4 model training.

The model training process uses a restart cosine annealing learning rate recession method, which causes the loss function to change due to the increase in the learning rate during the learning rate restart. In this paper, YOLOv4_GECA_3_SGDR_4 indicates that four restart learning rate operations were performed during the training of the YOLOv4_GECA_3 model, and so on. The YOLOv4_GECA_3_SGDR_4 model training process is illustrated in Fig. 17 with a large fluctuation in the classification loss function during the learning rate restart, followed by a continued decrease in the loss function.

The YOLOv4_GECA_3 model with a restart cosine annealing learning rate decay method introduced during the training process was tested on the test set after the training was completed, and the test results are shown in Table 3. Table 3 contains the test results of the YOLOv4 model with parameters for the improved model YOLOv4_GECA with a different number of restart learning rates and the YOLOv4_GECA_3_Decay network with exponential decay of learning rates. The results show that a restart cosine annealing learning rate decay approach is superior to the exponential decay approach on the YOLOv4_GECA model. From the table, we can see that the YOLOv4_GECA_3_SGDR_1 model has the highest mAP@0.5 and Recall index, where the average precision of 0.901 is 0.6% higher than YOLOv4_GECA_3, and the Recall of 0.907 is 1.3% higher than YOLOv4_GECA_3. The triple restart learning rate also brings a large improvement in model performance, with a mAP@0.5 of 0.898 compared to YOLOv4_GECA_3, and a Recall of 0.899 compared to YOLOv4_GECA_3, an improvement of 0.5%. Taken together, the introduction of a restart cosine annealing learning rate decay approach during training is effective and does not impose any burden on the model parameters, and has no effect on the detection speed of the model.

**Table 3:** Performance comparison of different restart learning rates of YOLOv4_GECA_3_SGDR.

Model	mAP@0.5	Recall	Parameters(× 10^6^)	Fps
YOLOv4	0.885	0.888	63.9592	35.77
YOLOv4_GECA_3	0.895	0.894	63.9593	30.07
YOLOv4_GECA_3_Decay	0.889	0.887	63.9593	30.04
YOLOv4_GECA_3_SGDR_1	0.901	0.907	63.9593	30.31
YOLOv4_GECA_3_SGDR_2	0.896	0.892	63.9593	30.05
YOLOv4_GECA_3_SGDR_3	0.898	0.899	63.9593	30.67
YOLOv4_GECA_3_SGDR_4	0.897	0.895	63.9593	30.41

### Experiment summary

**Figure 18:**
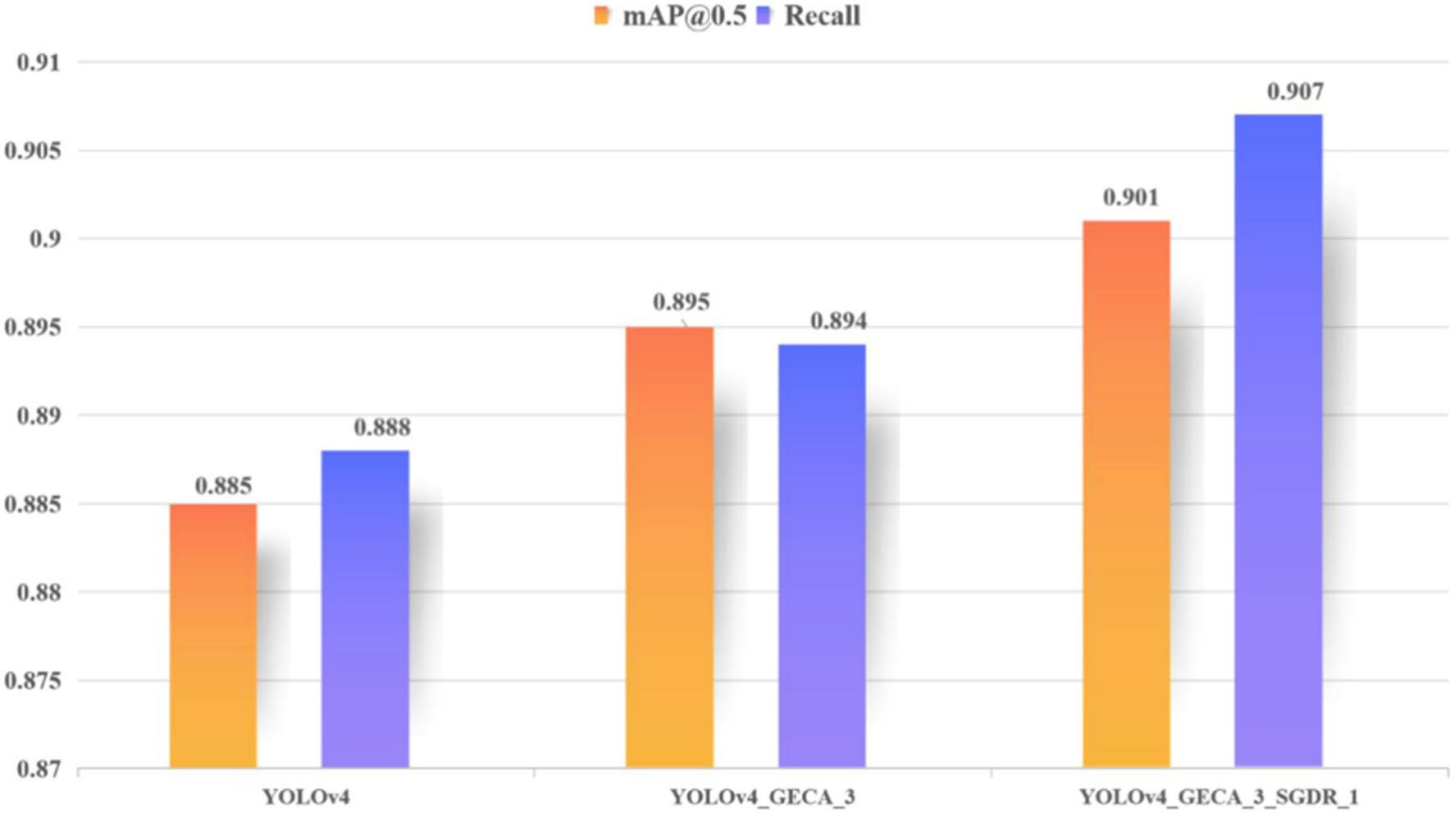
Comparison of model performance.

The foreign body detection model in this study is trained on the self-made mining belt foreign body image dataset, and the experimental comparison shows that the YOLOv4 model performs better in terms of accuracy and Recall compared with the YOLOv3, YOLOv3-spp, and YOLOv5 models, with an mAP of 0.885 and a Recall of 0.888, so the YOLOv4 model is chosen as the base model for foreign body detection, and improve the structure of YOLOv4 model and the way of decaying learning rate during training, respectively. Firstly, the YOLOv4 model structure is improved, and the YOLOv4_GECA model is built in the backbone network CSPDarknet53 by combining the GECA attention mechanism proposed in this paper. By training and testing the YOLOv4_GECA model with different hyperparameters k separately, the best performance of the YOLOv4_GECA_3 model is obtained. Next, the learning rate decay method during the training of the YOLOv4_GECA_3 model is improved, and the restart cosine annealing learning rate decay method is proposed to further improve the performance of the model. YOLOv4_GECA_3_SGDR_1 has the best performance with an mAP of 0.901 and a Recall of 0.907. Figure 18 shows the performance comparison of the YOLOv4, YOLOv4_GECA_3, and YOLOv4_GECA_3_SGDR_1 models, and we can see that mAP and Recall of the YOLOv4 model are improved after combining the GECA attention mechanism proposed in this paper. The model performance is further improved by the decay method. The model improvement approach in this study improves the foreign body performance of the model, reduces the rate of missed detection and false detection, and can still be applied in the actual production process although there is a decrease in the detection speed.

For different foreign body recognition, Tables 4 and 5 show the results of YOLOv4, YOLOv4_GECA_3, and YOLOv4_GECA_3_SGDR_1 models for each type of foreign body recognition, respectively. From Table 4, it can be seen that the two improved models YOLOv4_GECA and YOLOv4_GECA_SGDR_1 proposed in this paper improve the detection accuracy of each category of foreign bodies compared to the YOLOv4 base model, for example, the mAP@0.5 of YOLOv4 model in steel cable foreign body detection is only 0.638, while YOLOv4_GECA_3 improves this index to 0.664. Also from Table 5, it can be seen that the improved model in this paper improves the Recall for each category of foreign body detection, YOLOv4 has a Recall of 0.957 for foreign body detection in plastic pipes, while YOLOv4_GECA_3 improves the index to 0.975. The results from these two tables show that the GECA attention mechanism proposed in this paper has improved the detection of difficult foreign bodies caused by small objects, partial occlusion of foreign bodies, and dust interference, proving the effectiveness of the GECA attention mechanism.

**Table 4:** Improvement model comparison of mAP@0.5 indicators for various categories of foreign bodies.

Model	Steel bars	Plastic pipes	Wood	Iron	Steel cables
YOLOv4	0.861	0.965	0.964	0.995	0.638
YOLOv4_GECA_3	0.872	0.975	0.969	0.996	0.664
YOLOv4_GECA_3_SGDR_1	0.879	0.973	0.968	0.995	0.690

**Table 5:** Improvement model comparison of Recall indicators for various categories of foreign bodies.

Model	Steel bars	Plastic pipes	Wood	Iron	Steel cables
YOLOv4	0.837	0.957	0.962	0.980	0.702
YOLOv4_GECA_3	0.841	0.976	0.970	0.982	0.702
YOLOv4_GECA_3_SGDR_1	0.848	0.968	0.979	0.986	0.753

**Figure 19:**
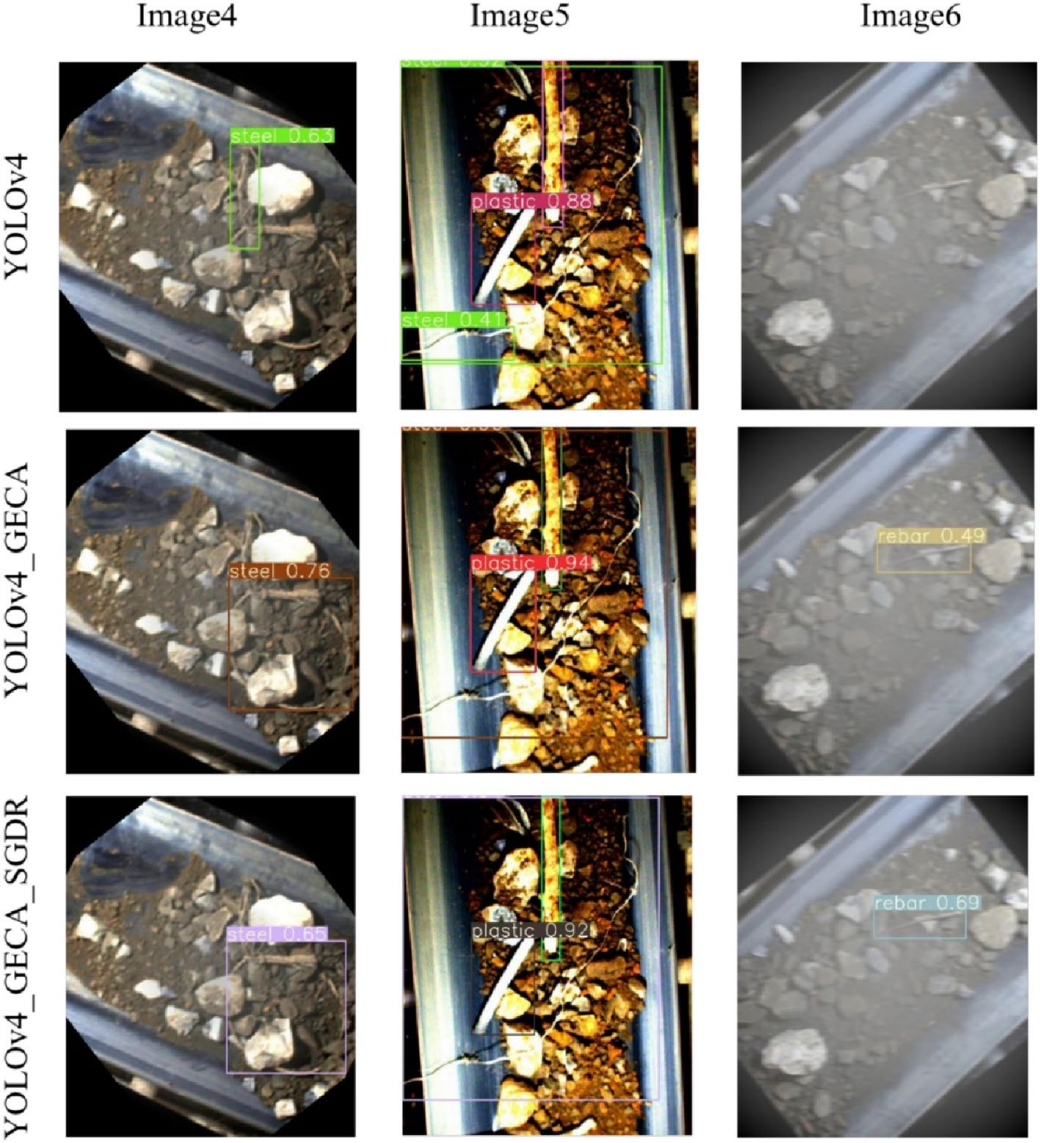
Comparison of the detection effect of the three models.

A comparison of the prediction results of YOLOv4, YOLOv4_GECA, and YOLOv4_GECA_SGDR models for some images is shown in Fig. 19. Three images containing foreign bodies are shown from left to right, and the detection results of three object detection models for foreign body images are shown in order from top to bottom. In the Image4 image, it can be seen that the steel cable foreign body is partially obscured and the YOLOv4 model deviates in predicting the foreign body location compared to the other two improved models, and the improved models YOLOv4_GECA and YOLOv4_GECA_SGDR in this paper are more accurate in detecting the foreign body. In the Image5 image, influenced by light and occlusion, the YOLOv4 model experienced a false positive, predicting a single steel cable foreign body as two, and the plastic tube prediction confidence was lower than the other two improved models. In the Image6 image, due to the buried and relatively small rebar, which was disturbed by dust, light, and obscuration of many factors, the YOLOv4 model experienced a missed rebar foreign body detection, which would have an impact on the subsequent production safety. The comparison of image detection results shows that the two improved models YOLOv4_GECA and YOLOv4_GECA_SGDR proposed in this paper are more effective than the YOLOv4 base model in detecting small objects, foreign bodies partially obscured and foreign bodies difficult to identify due to dust interference. In summary, the proposed improved YOLOv4_GECA model with GECA-based attention mechanism and the proposed YOLOv4_GECA_SGDR model with decaying restart cosine annealing learning rate is validated utilizing comparative experiments to improve the detection accuracy and detection recall in the detection of difficult-to-identify foreign bodies caused by small objects, foreign bodies being partially obscured and dust interference, and can be applied to the actual site of ore transportation.

## Conclusion

This paper selects YOLOv4 as the basic foreign object detection model and proposes the GECA attention mechanism to build the YOLOv4_GECA model, which can fully exploit the global context information of the feature map, obtain the dependency relationship between each channel, and improve the feature extraction ability of the model for small target foreign objects, partially occluded foreign objects and dust interference images. It is also proposed to improve the original learning rate decay method of YOLOv4 by constructing a restart cosine annealing learning rate decay method based on the YOLOv4_GECA model with the YOLOv4_GECA_SGDR network. This network increases the versatility of the foreign object detection network training and optimization process and improves the performance of the model in detecting foreign object images without increasing the burden of the model, which is more suitable for the training of foreign object detection models for mining belts. Experiments were conducted on the created mining belt foreign body detection dataset, and the results of the experimental analysis showed that the accuracy and recall of the YOLOv4_GECA method proposed in this paper were significantly improved and achieved a balance of accuracy and speed, where the accuracy reached 0.901, the recall 0.907, and the Fps 30.31 ms, achieving the purpose of improving the detection of foreign bodies in mineral belts.

## Supplementary Information

Supplementary Information.

## Data Availability

The datasets used and/or analysed during the current study available from the corresponding author on reasonable request.

## References

[1] Janssens E, De Beenhouwer J, Van Dael M (2018). Neural network Hilbert transform-based filtered back projection for fast inline x-ray inspection. Meas. Sci. Technol..

[2] Ren S, He K, Girshick R, et al. Faster r-CNN: Towards real-time object detection with region proposal networks. *Adv. Neural Inf. Process. Syst.* 28 (2015). 10.1109/TPAMI.2016.2577031 27295650

[3] Redmon, J., Divvala, S., Girshick, R., et al. You only look once: Unified, real-time object detection. in *2016 IEEE Conference on Computer Vision and Pattern Recognition*, Las Vegas, USA, 2016: 779–788. doi: 10.1109/CVPR.2016. 91.

[4] Redmon, J. and Farhadi, A. YOLO9000: Better, faster, stronger. in *2017 IEEE Conference on Computer Vision and Pattern Recognition*, Honolulu, USA, 2017: 6517–6525. doi: 10.1109/CVPR.2017.690.

[5] Redmon, J. and Farhadi, A. YOLOv3: An incremental improvement. arXiv preprint arXiv:1804.02767, (2018).

[6] Bochkovskiy A, Wang CY, Liao HYM. YOLOv4: Optimal speed and accuracy of object detection. arXiv: 2004.10934, (2020).

[7] Liu W, Anguelov D, Erhan D, et al. SSD: Single Shot MultiBox detector. in *Proceedings of the 14th European Conference on Computer Vision*. Amsterdam: Springer, 2016. 21–37.

[8] Jocher, G., Stoken, A., Borovec, J., et al. Ultralytics/YOLOv5: V3.1 - bug fixes and performance improvements[EB/OL]. 10.5281/zenodo.4154370 (2020).

[9] Li, C., Li, L., Jiang, H., et al. YOLOv6: A single-stage object detection framework for industrial applications. arXiv preprint arXiv:2209.02976, (2022).

[10] Wang, C. Y., Bochkovskiy, A., Liao, H. Y. M. YOLOv7: Trainable bag-of-freebies sets new state-of-the-art for real-time object detectors. arXiv preprint arXiv:2207.02696, (2022).

[11] Cao X, Wang P, Meng C (2018). Region-based CNN for foreign object debris detection on airfield pavement. Sensors.

[12] Xu H, Han Z, Feng S (2018). Foreign object debris material recognition based on convolutional neural networks. J. Image Video Proc..

[13] Rong D, Xie L, Ying Y (2019). Computer vision detection of foreign objects in walnuts using deep learning. Comput. Electron. Agric..

[14] He Q, Yao Z, Jiang Z, Chen Y, Deng J, Xiang W (2019). Detection of foreign matter on high-speed train underbody based on deep learning. IEEE Access.

[15] Pang L, Liu H, Chen Y (2020). Real-time concealed object detection from passive millimeter wave images based on the YOLOv3 algorithm. Sensors.

[16] Chen Y, Sun X, Xu L (2022). Application of YOLOv4 algorithm for foreign object detection on a belt conveyor in a low-illumination environment. Sensors.

[17] Qiu Z, Zhao Z, Chen S (2022). Application of an improved YOLOv5 algorithm in real-time detection of foreign objects by ground penetrating radar. Remote Sensing.

[18] Jing Y, Zheng H, Lin C (2022). Foreign object debris detection for optical imaging sensors based on random forest. Sensors.

[19] Abramson HG, Curry EJ, Mess G (2022). Automatic detection of foreign body objects in neurosurgery using a deep learning approach on intraoperative ultrasound images: From animal models to first in-human testing. Front. Surg..

[20] Sowmya V, Radha R. Heavy-Vehicle Detection Based on YOLOv4 featuring Data Augmentation and Transfer-Learning Techniques. in *Journal of Physics: Conference Series*. Vol 1911, 1, 012029 (IOP Publishing, 2021).

[21] Hou Z, Liu X, Chen L. Object detection algorithm for improving non-maximum suppression using GIoU.in *IOP Conference Series: Materials Science and Engineering*. Vol. 720, no: 1, 012062 (IOP Publishing, 2020).

[22] Wang C Y, Liao H Y M, Wu Y H, et al. CSPNet: A new backbone that can enhance learning capability of CNN. in *Proceedings of the IEEE/CVF conference on computer vision and pattern recognition workshops*. 390–391 (2020).

[23] Ge R, Shen T, Zhou Y (2021). Convolutional squeeze-and-excitation network for ECG arrhythmia detection. Artif. Intell. Med..

