# Supplementary material for: Mining belt foreign body detection method based on YOLOv4_GECA model
Source: Sci Rep. 2023 Jun 1;13:8881. doi: 10.1038/s41598-023-35962-3 (PMC10235127; doi:10.1038/s41598-023-35962-3)

Mining Belt Foreign Body Detection Method Based on YOLOv4_GECA Model

Dong Xiao[1,2,*]·Panpan Liu[1,2]·Jichun Wang[3,4]·Zhengmin Gu[5]·Hang Yu[1]

Dong Xiao
xiaodong@ise.neu.edu.cn
Panpan Liu
l2206979789@163.com
Jichun Wang
Sophiewjc@163.com
Zhengmin Gu
guzhengmin@cmu1h.com
Hang Yu
1311582776@qq.com

[1] Information Science and Engineering School, Northeastern University, Shenyang 110004, China

[2] Liaoning Key Laboratory of Intelligent Diagnosis and Safety for Metallurgical Industry, Northeastern University, Shenyang 110819, China

[3] Shenyang Institute of Computing technology Co. Ltd. , CAS, Shenyang, Liaoning 110168, China

[4] College of Science, Shenyang Jianzhu University, Shenyang, Liaoning 110168, China

[5] The First Hospital Of China Medical University, Shenyang, China

These images below are some of the images in the dataset.

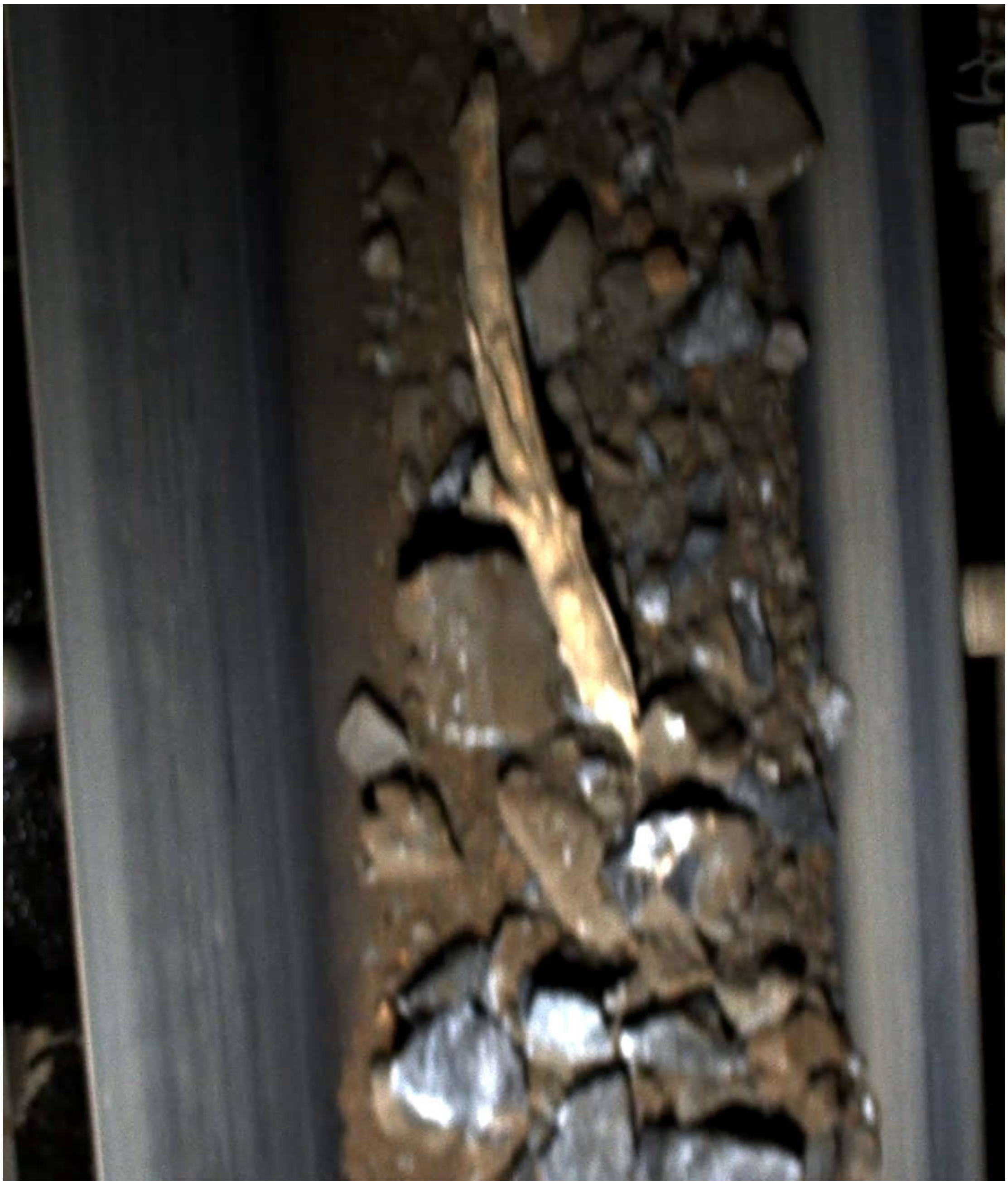

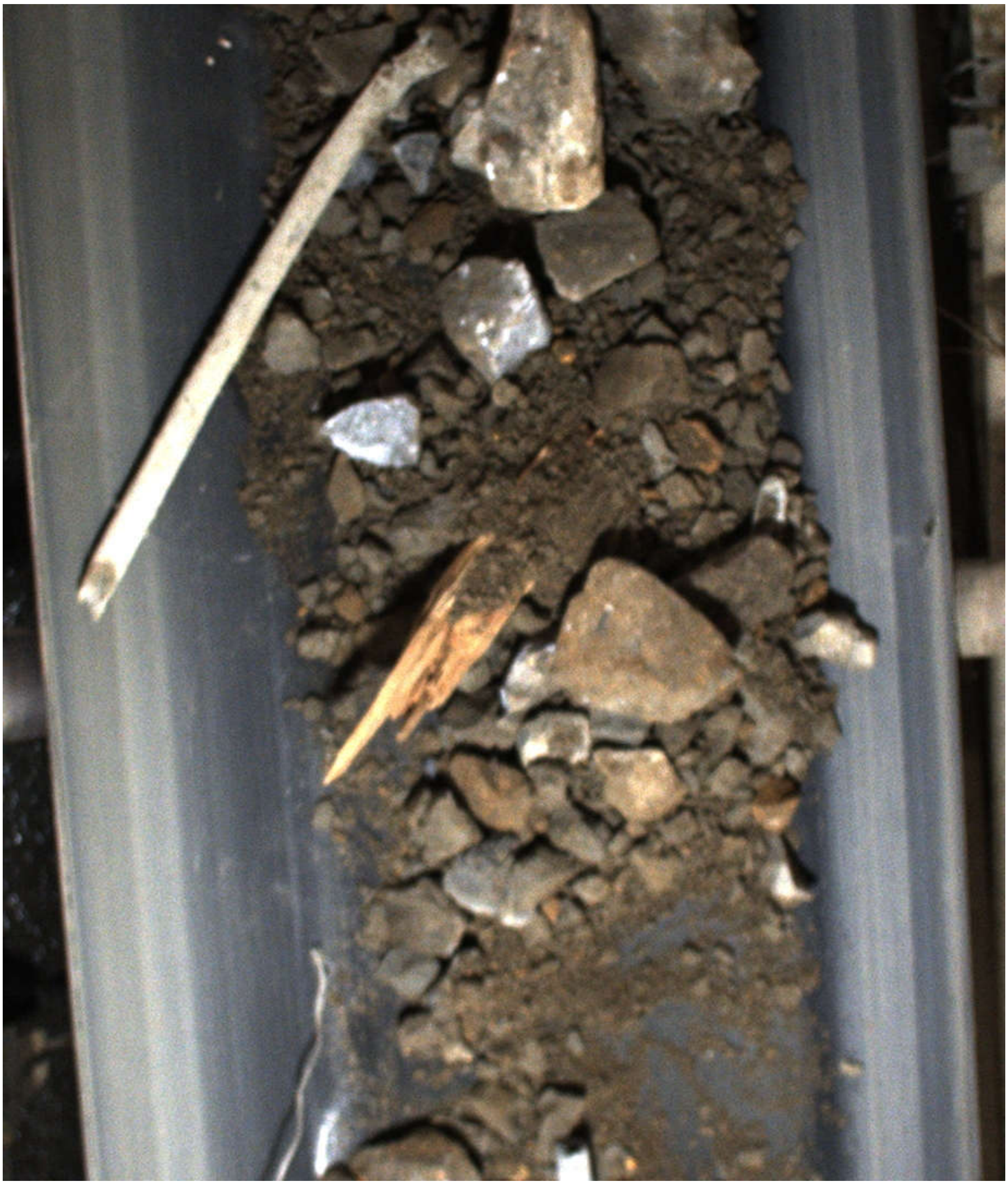

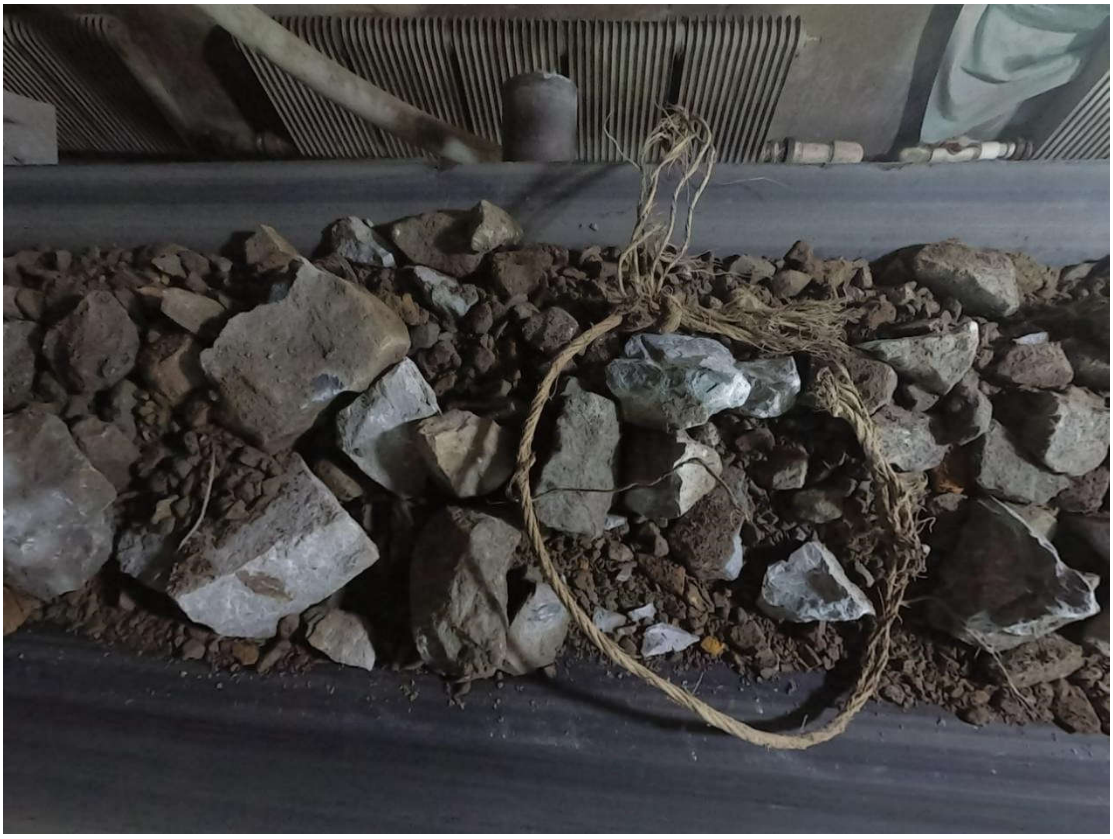

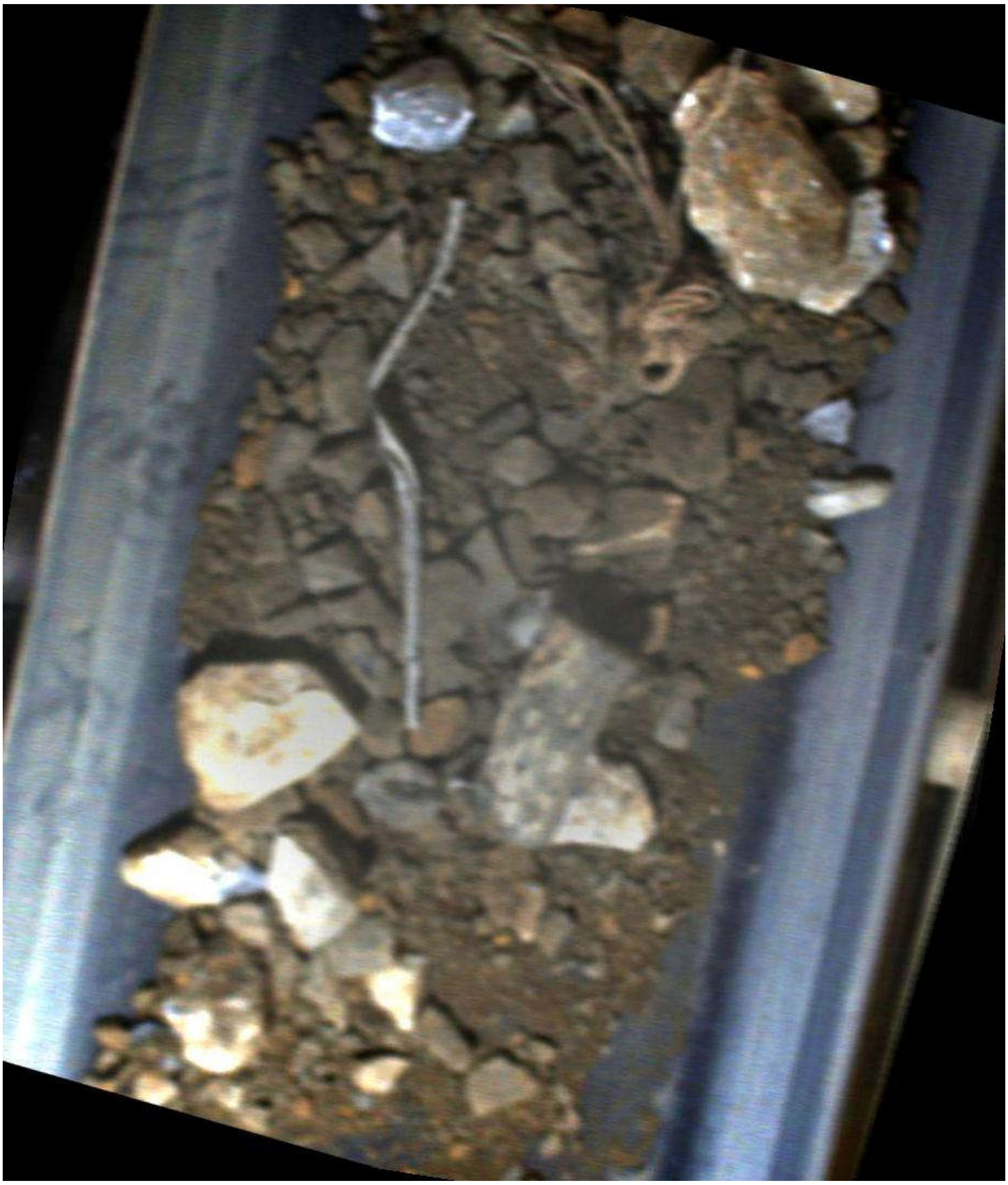

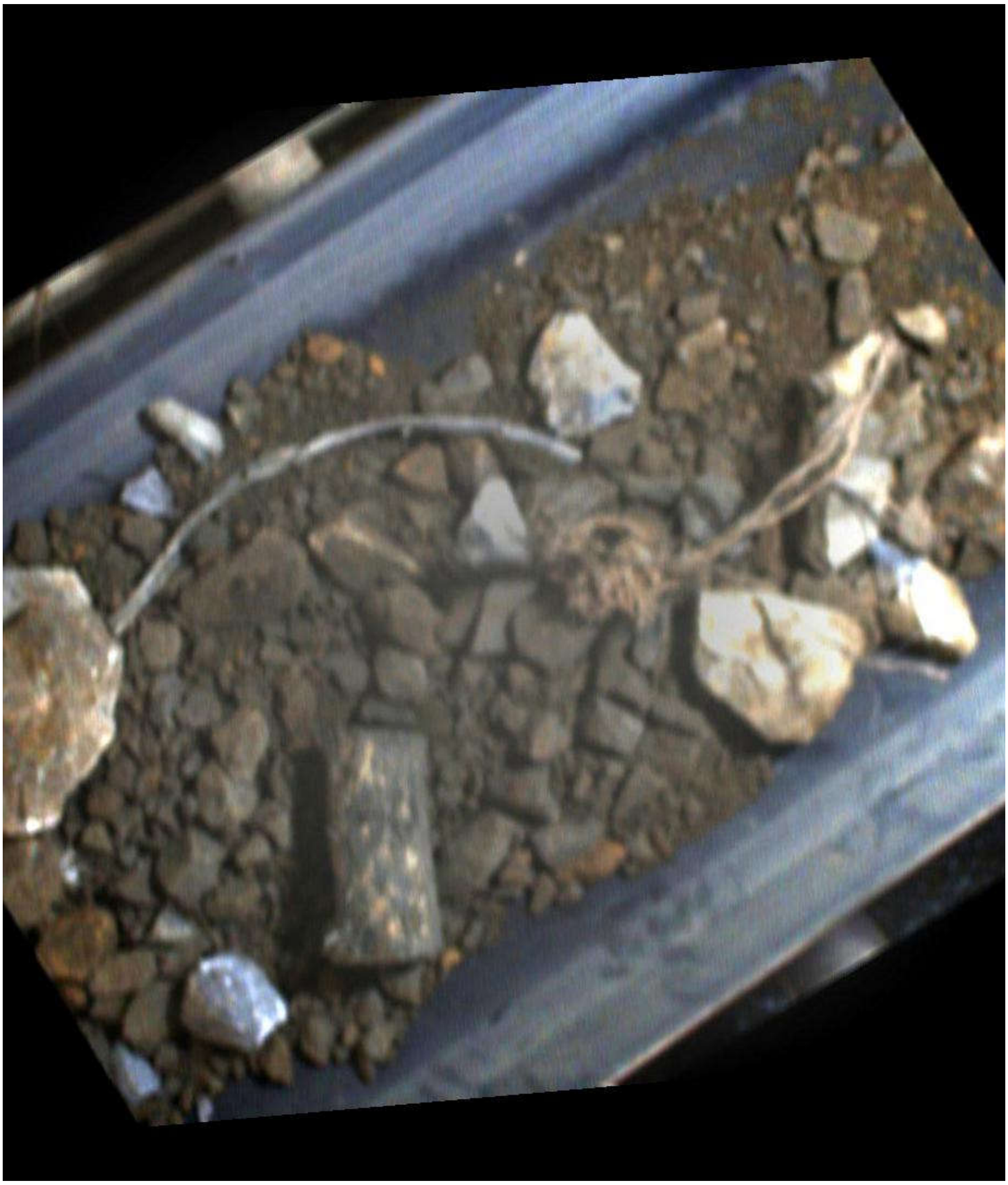

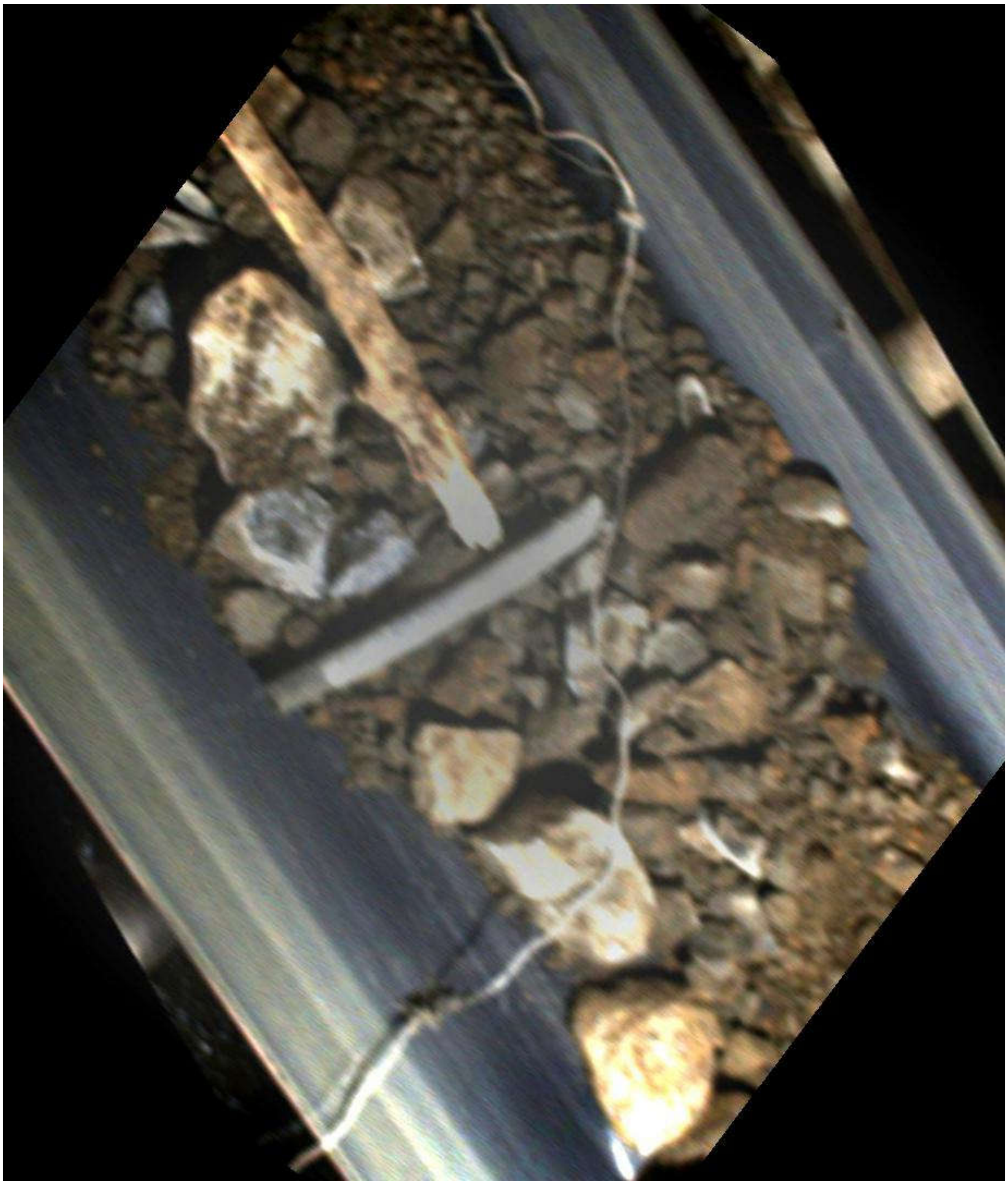

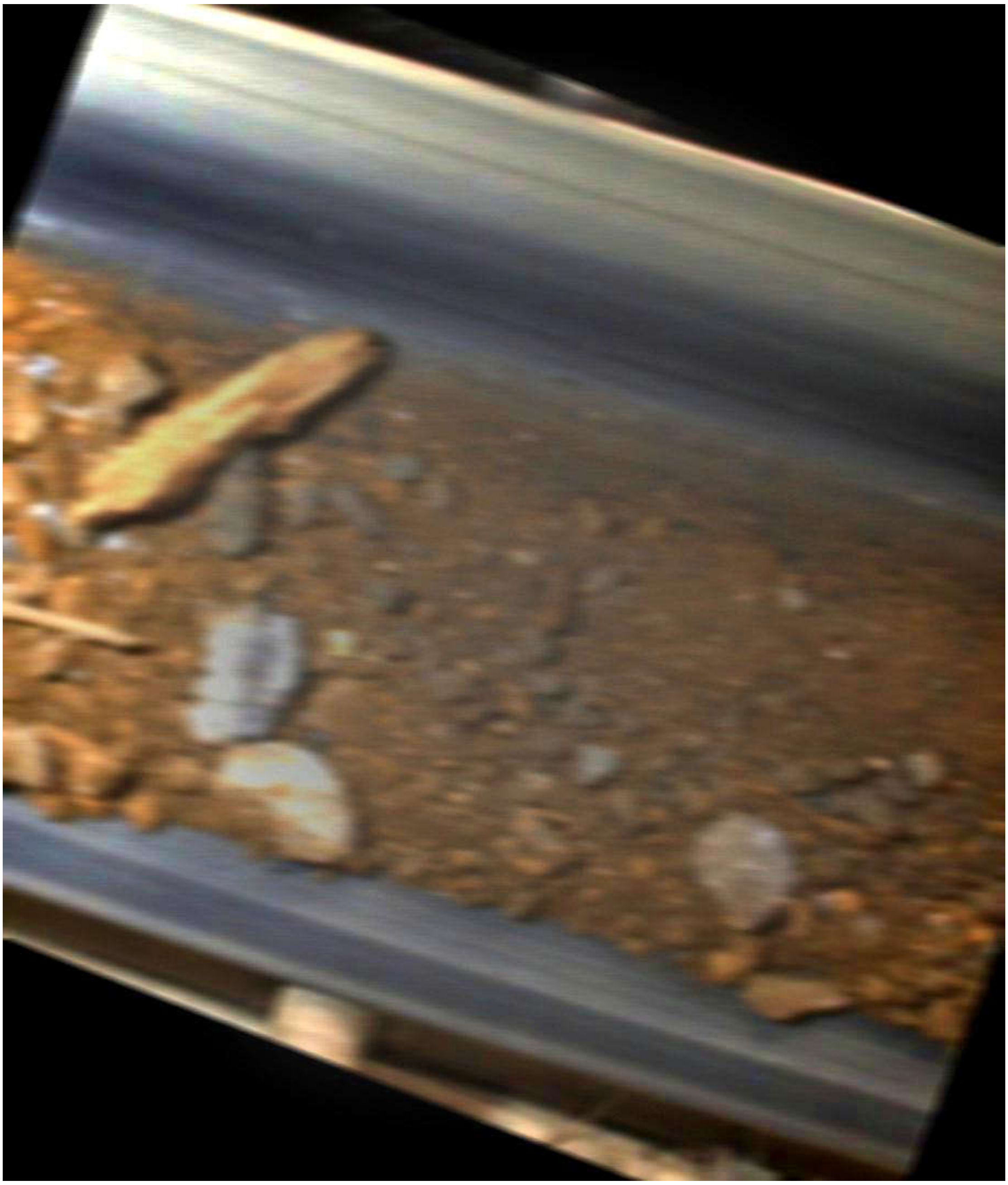

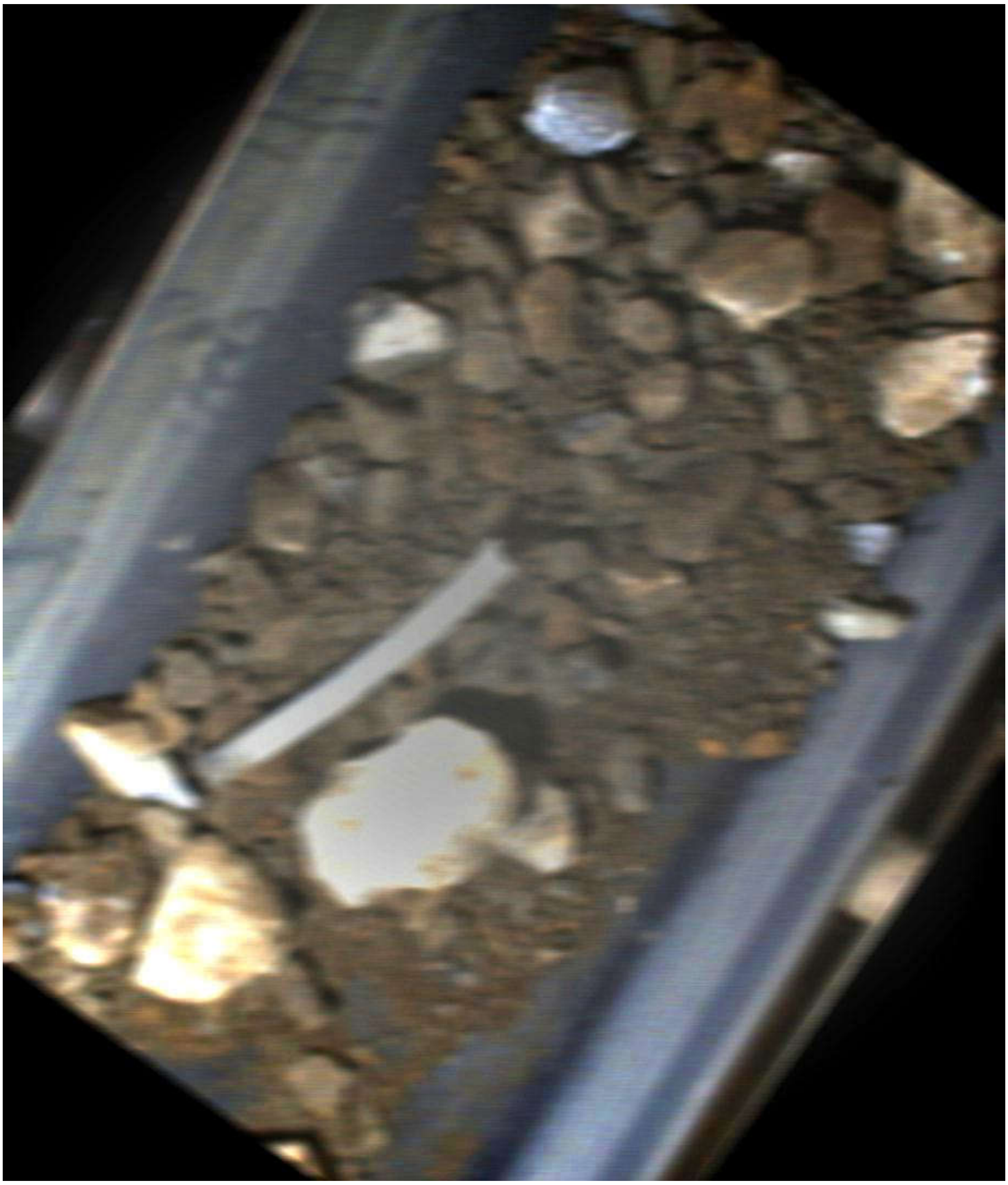

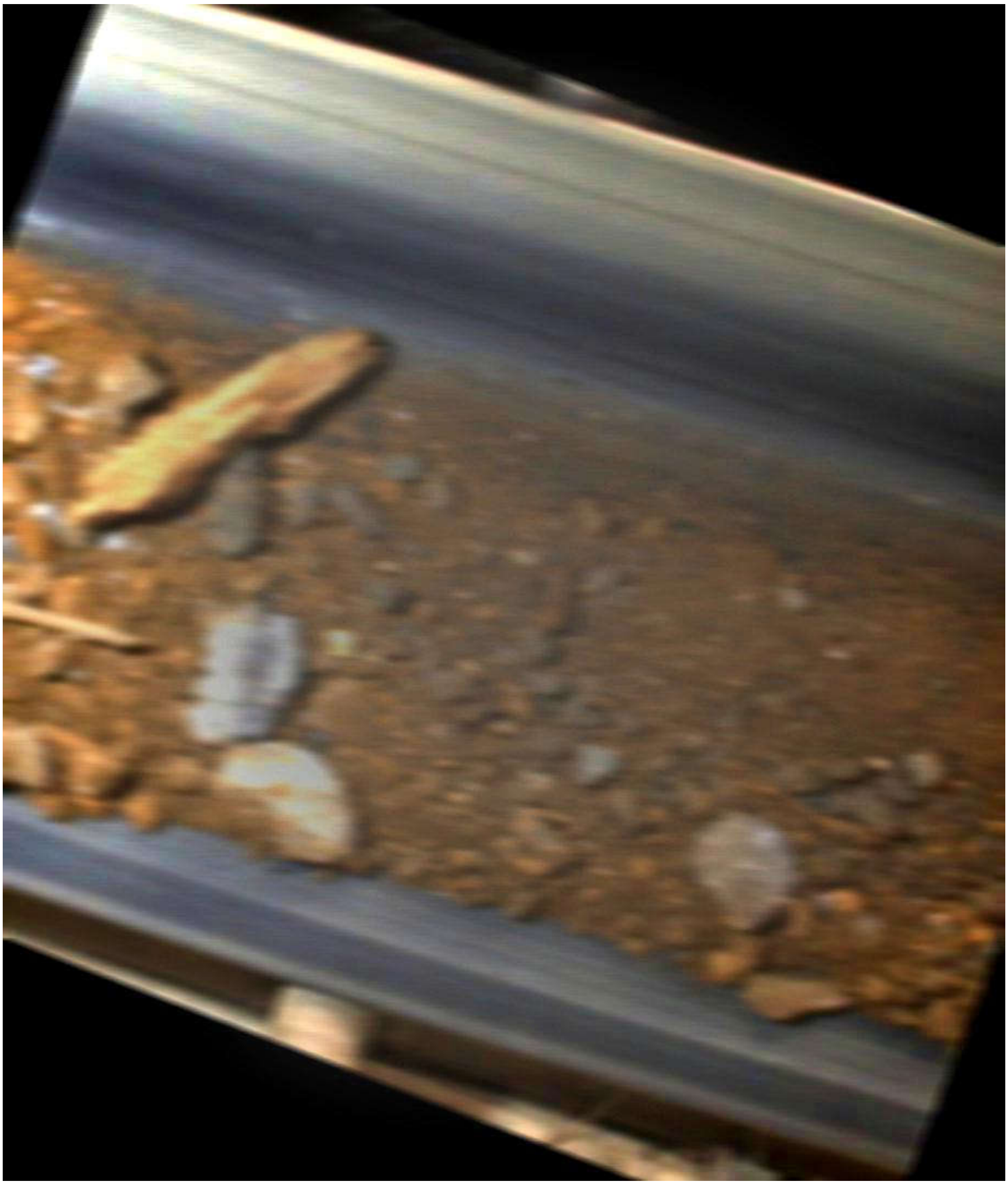

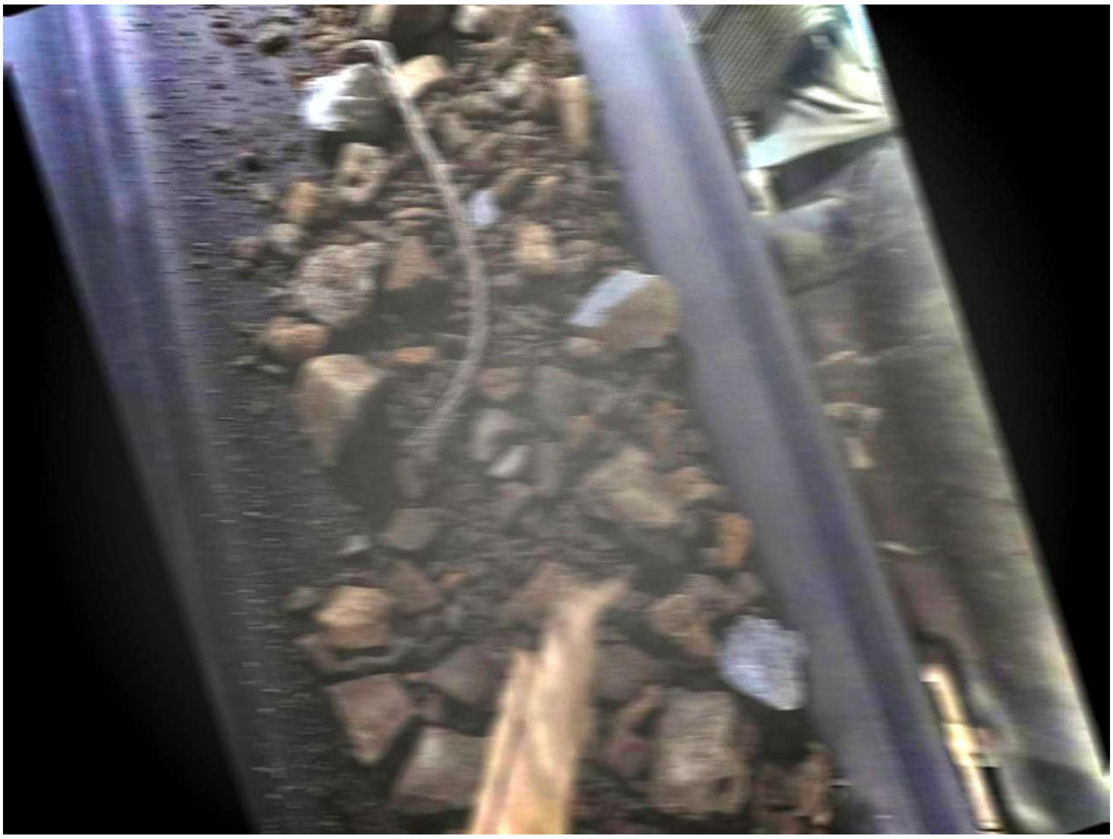

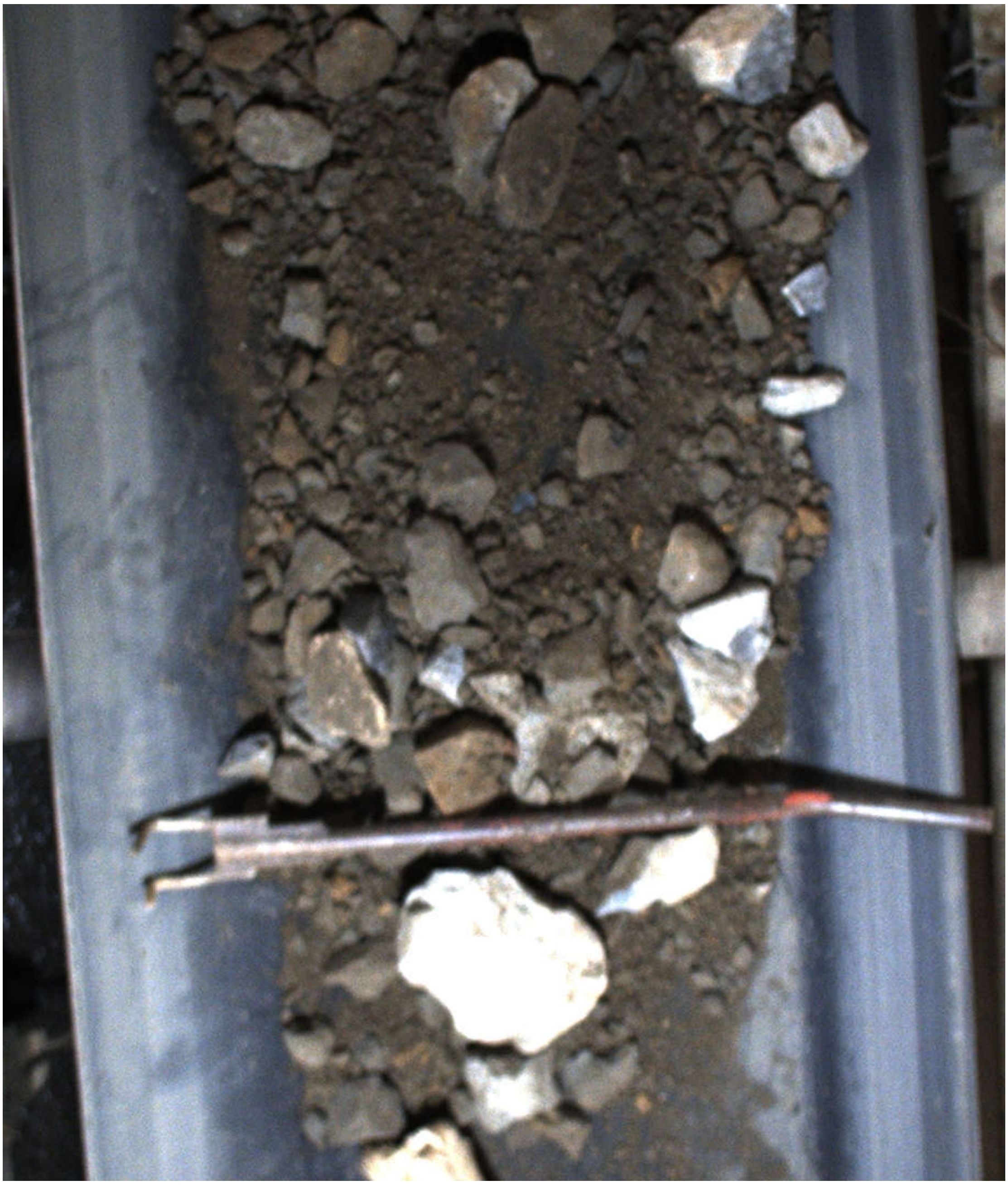

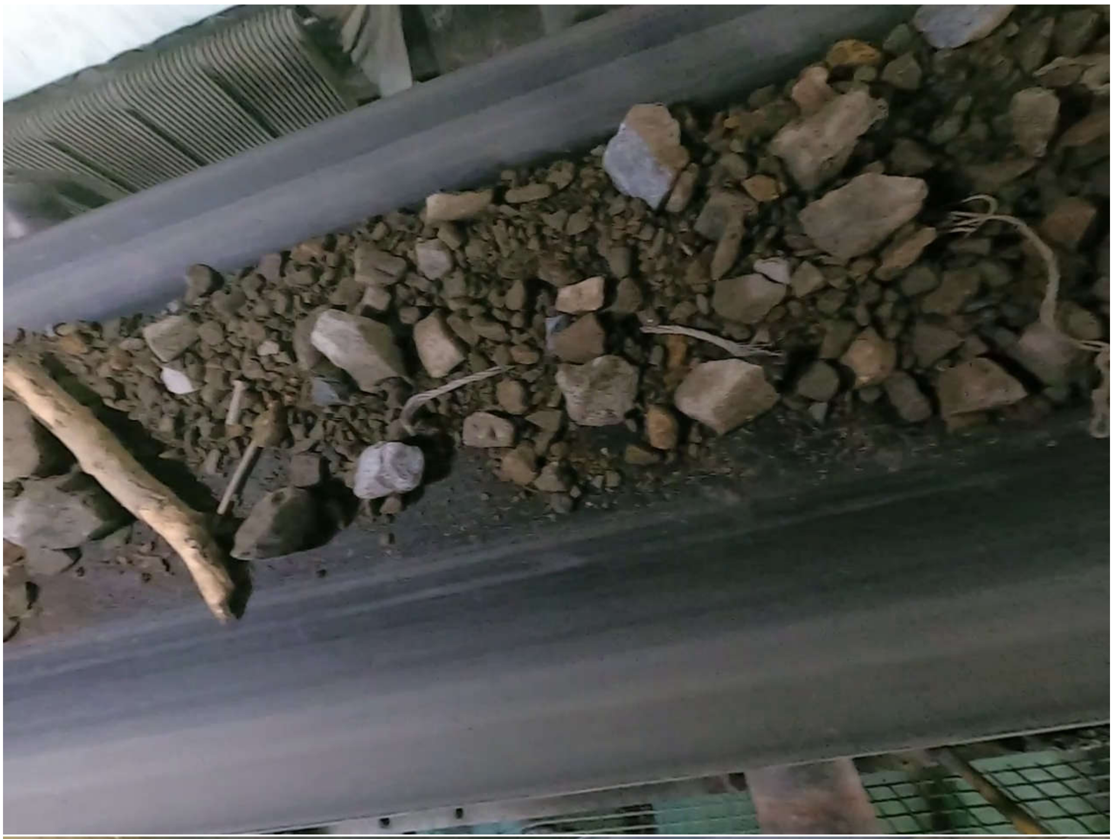

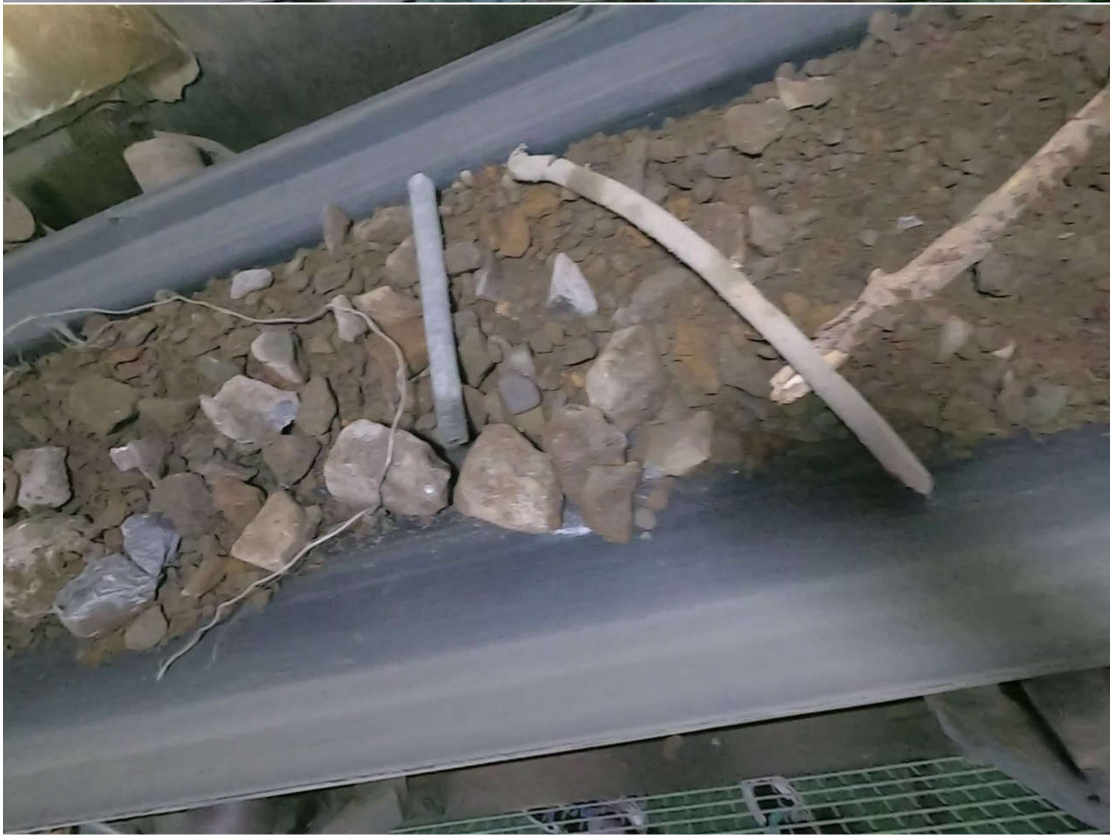

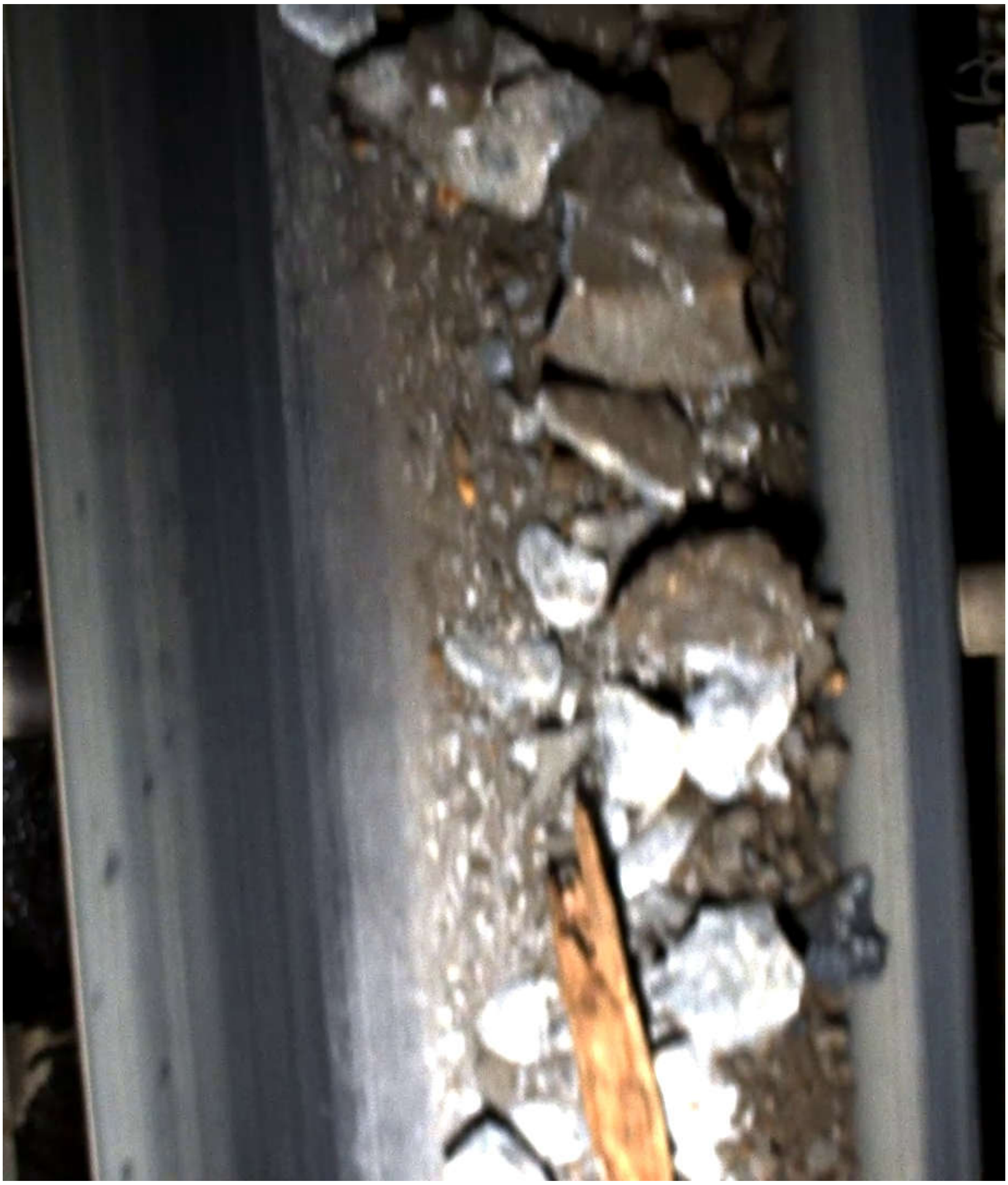

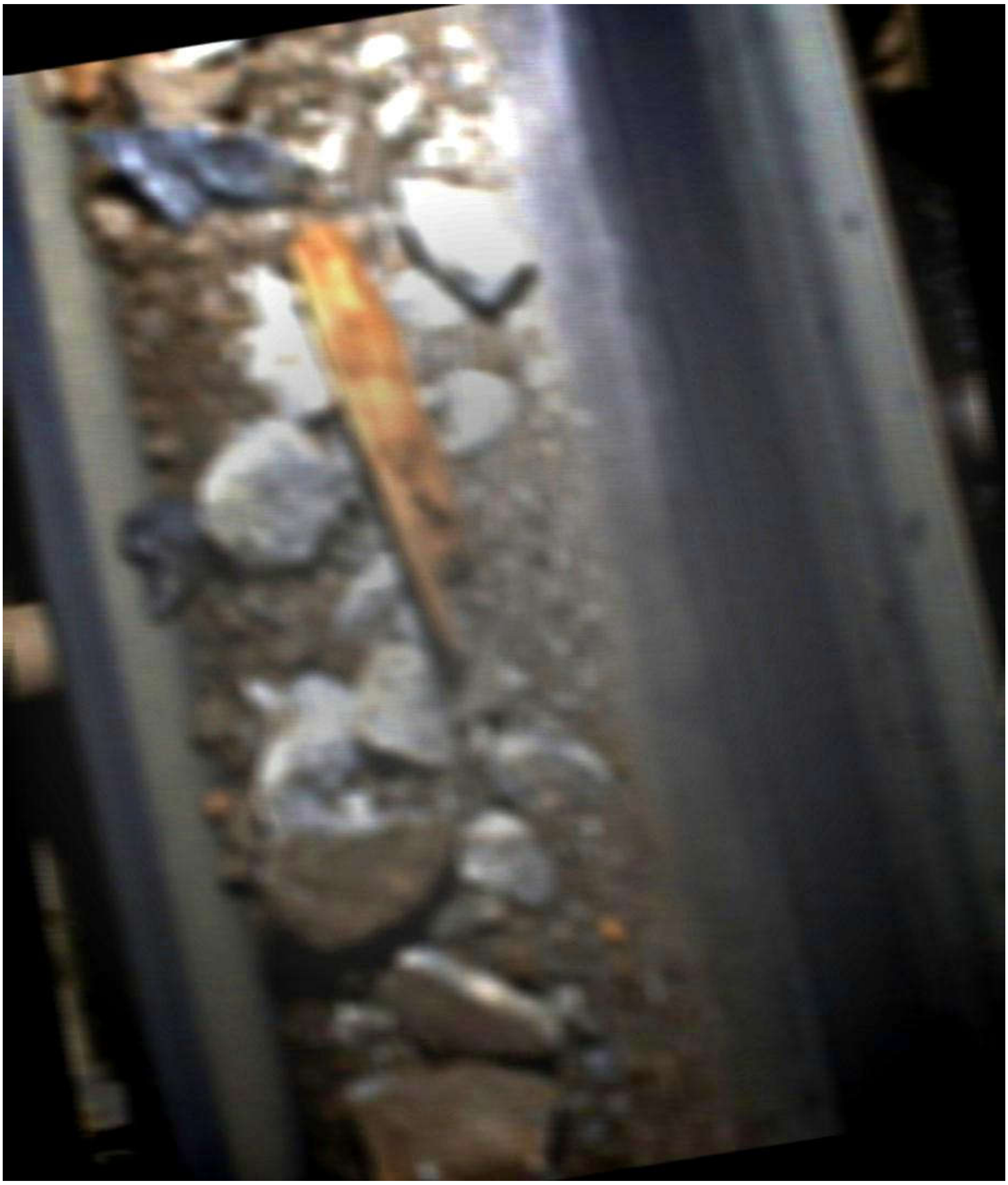

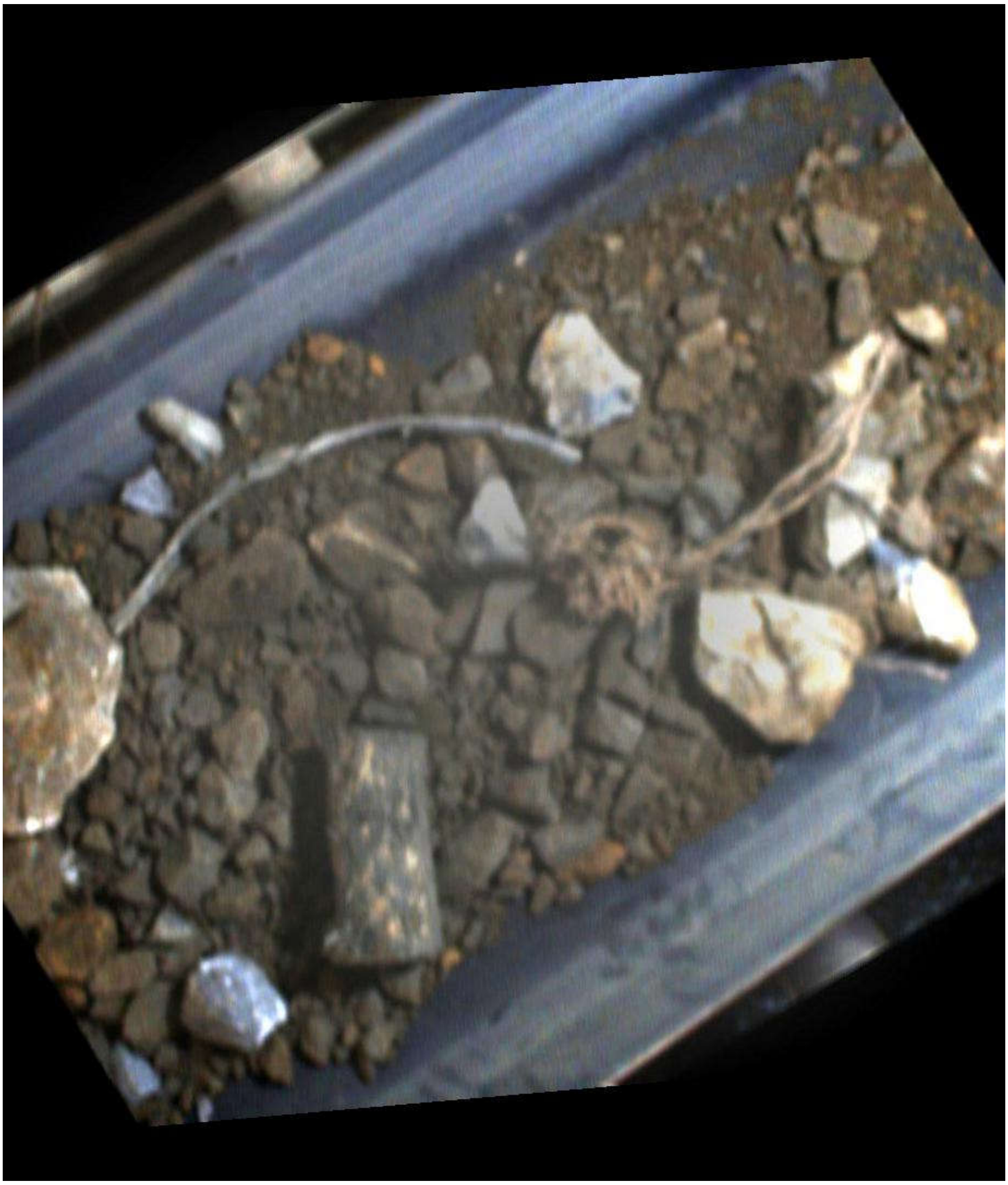

Supplement: Supplementary file 1 — Supplementary Information. [file 41598_2023_35962_MOESM1_ESM.pdf]
